# Competing risk events in antimalarial drug trials in uncomplicated *Plasmodium falciparum* malaria: a WorldWide Antimalarial Resistance Network individual participant data meta-analysis

**DOI:** 10.1186/s12936-019-2837-4

**Published:** 2019-07-05

**Authors:** The WorldWide Antimalarial Resistance Network Methodology Study Group, Prabin Dahal, Julie Anne Simpson, Salim Abdulla, Jane Achan, Ishag Adam, Aarti Agarwal, Richard Allan, Anupkumar R. Anvikar, Emmanuel Arinaitwe, Elizabeth A. Ashley, Ghulam Rahim Awab, Quique Bassat, Anders Björkman, Francois Bompart, Steffen Borrmann, Teun Bousema, Ingrid Broek, Hasifa Bukirwa, Verena I. Carrara, Marco Corsi, Michel Cot, Umberto D’Alessandro, Timothy M. E. Davis, Marit de Wit, Philippe Deloron, Meghna Desai, Pedro Rafael Dimbu, Djibrine Djalle, Abdoulaye Djimde, Grant Dorsey, Ogobara K. Doumbo, Chris J. Drakeley, Stephan Duparc, Michael D. Edstein, Emmanuelle Espie, Abul Faiz, Catherine Falade, Caterina Fanello, Jean-Francois Faucher, Babacar Faye, Filomeno de Jesus Fortes, Nahla B. Gadalla, Oumar Gaye, J. Pedro Gil, Brian Greenwood, Anastasia Grivoyannis, Kamal Hamed, Tran Tinh Hien, David Hughes, Georgina Humphreys, Jimee Hwang, Maman Laminou Ibrahim, Bart Janssens, Vincent Jullien, Elizabeth Juma, Erasmus Kamugisha, Corine Karema, Harin A. Karunajeewa, Jean R. Kiechel, Fred Kironde, Poul-Erik Kofoed, Peter G. Kremsner, Valerie Lameyre, Sue J. Lee, Kevin Marsh, Andreas Mårtensson, Mayfong Mayxay, Hervé Menan, Petra Mens, Theonest K. Mutabingwa, Jean-Louis Ndiaye, Billy E. Ngasala, Harald Noedl, Francois Nosten, Andre Toure Offianan, Mary Oguike, Bernhards R. Ogutu, Piero Olliaro, Jean Bosco Ouedraogo, Patrice Piola, Christopher V. Plowe, Mateusz M. Plucinski, Oliver James Pratt, Zulfikarali Premji, Michael Ramharter, Christophe Rogier, Lars Rombo, Philip J. Rosenthal, Patrick Sawa, Birgit Schramm, Carol Sibley, Veronique Sinou, Sodiomon Sirima, Frank Smithuis, Sarah G. Staedke, Inge Sutanto, Ambrose Otau Talisuna, Joel Tarning, Walter R. J. Taylor, Emmanuel Temu, Kamala L. Thriemer, Nhien Nguyen Thuy, Venkatachalam Udhayakumar, Johan Ursing, Michel van Herp, Michele van Vugt, Christopher Whitty, Yavo William, Cornelis Winnips, Issaka Zongo, Philippe Guerin, Ric N. Price, Kasia Stepniewska

**Affiliations:** WorldWide Antimalarial Resistance Network (WWARN), Centre for Tropical Medicine and Global Health, Nuffield Department of Clinical Medicine, University of Oxford, Oxford, UK

**Keywords:** *Plasmodium falciparum*, Treatment efficacy study, Competing risk event

## Abstract

**Background:**

Therapeutic efficacy studies in uncomplicated *Plasmodium falciparum* malaria are confounded by new infections, which constitute competing risk events since they can potentially preclude/pre-empt the detection of subsequent recrudescence of persistent, sub-microscopic primary infections.

**Methods:**

Antimalarial studies typically report the risk of recrudescence derived using the Kaplan–Meier (K–M) method, which considers new infections acquired during the follow-up period as censored. Cumulative Incidence Function (CIF) provides an alternative approach for handling new infections, which accounts for them as a competing risk event. The complement of the estimate derived using the K–M method (1 minus K–M), and the CIF were used to derive the risk of recrudescence at the end of the follow-up period using data from studies collated in the WorldWide Antimalarial Resistance Network data repository. Absolute differences in the failure estimates derived using these two methods were quantified. In comparative studies, the equality of two K–M curves was assessed using the log-rank test, and the equality of CIFs using Gray’s *k*-sample test (both at 5% level of significance). Two different regression modelling strategies for recrudescence were considered: cause-specific Cox model and Fine and Gray’s sub-distributional hazard model.

**Results:**

Data were available from 92 studies (233 treatment arms, 31,379 patients) conducted between 1996 and 2014. At the end of follow-up, the median absolute overestimation in the estimated risk of cumulative recrudescence by using 1 minus K–M approach was 0.04% (interquartile range (IQR): 0.00–0.27%, Range: 0.00–3.60%). The overestimation was correlated positively with the proportion of patients with recrudescence [Pearson’s correlation coefficient (*ρ*): 0.38, 95% Confidence Interval (CI) 0.30–0.46] or new infection [*ρ*: 0.43; 95% CI 0.35–0.54]. In three study arms, the point estimates of failure were greater than 10% (the WHO threshold for withdrawing antimalarials) when the K–M method was used, but remained below 10% when using the CIF approach, but the 95% confidence interval included this threshold.

**Conclusions:**

The 1 minus K–M method resulted in a marginal overestimation of recrudescence that became increasingly pronounced as antimalarial efficacy declined, particularly when the observed proportion of new infection was high. The CIF approach provides an alternative approach for derivation of failure estimates in antimalarial trials, particularly in high transmission settings.

**Electronic supplementary material:**

The online version of this article (10.1186/s12936-019-2837-4) contains supplementary material, which is available to authorized users.

## Background

**Fig. 1:**
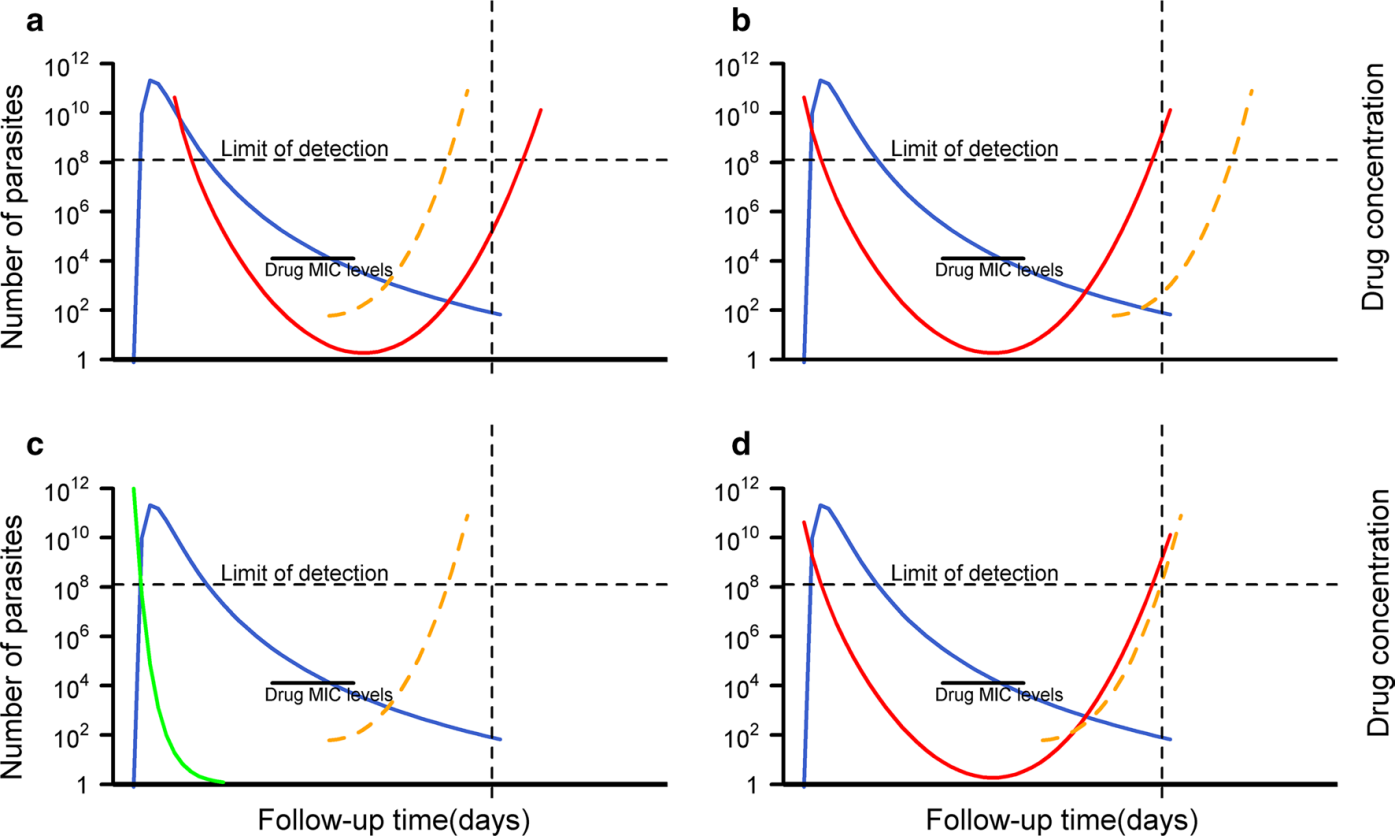
Situational competitiveness of newly emergent infections. Adapted from White-2002 [44]. The blue line represents a hypothetical drug concentration of partner component, the green and red lines represent scenarios for parasite burden versus time profiles following treatment for an infection where all the parasites are completely killed resulting in cure (green) and an infection where parasites are initially killed by high drug levels but with drug levels below the minimum inhibitory concentration (MIC), net parasite growth results in subsequent recrudescence (red). The orange line represents parasite-time profiles for a new infection. The left y-axis is for parasite density, and the right y-axis shows drug levels at hypothetical units. The vertical dotted line is the administrative end of the study follow-up. The horizontal dotted line represents the microscopic limit of detection for parasites. **a** Parasite population from a new inoculation out-competes the parasite population which caused the disease thus precluding recrudescence. In this situation, new infection is a biologically competing risk event. **b** In this situation new infection can be thought of as biologically competing risk event which doesn’t prevent recrudescence being observed. **c** The parasite population which caused the disease is completely eliminated. Here, new infection is not a competing event. **d** In this situation, the parasite population which caused the disease and which is derived from a novel inoculation appear at the same time

A competing risk is an event which precludes the occurrence of the primary event of interest [1]. The primary endpoint in therapeutic efficacy studies in uncomplicated *Plasmodium falciparum* malaria is recurrence of the parasite during the study follow-up which caused the original infection (recrudescence). Malaria recurrence may also be caused by a heterologous parasite, which can be either a newly acquired infection with *P. falciparum,* or another species of *Plasmodium*. In certain scenarios, such as when the parasite load of a newly acquired infection outnumbers the low level of parasitaemia of an existing infection, the recrudescent parasites may not be detected (Fig. 1a). In such a scenario, a new infection can pre-empt the patency of a recrudescent infection thereby constituting a competing risk event (Table 1).

**Table 1:** Possible outcomes in antimalarial studies of uncomplicated *P. falciparum* malaria

Possible outcomes	Description
Recrudescence of *P. falciparum*	Recurrent infection caused by the parasites that survived treatment. This is the primary endpoint in antimalarial efficacy studies of uncomplicated *P. falciparum* malaria
Novel infections due to *P. falciparum or P. vivax*	Recurrent infection due to a new parasite strain or strains during the follow-up. This can be due to a different *Plasmodium* species or due to the same species but different genotype from the initial parasite infection
Indeterminate recurrent infection	Recurrent infection with the same parasite species but in which molecular analysis is unable to discriminate between a recrudescent or new infection (e.g. due to unsuccessful amplification of DNA). These patients are usually excluded when deriving efficacy estimates
Patients in whom follow-up is curtailed before the end of the study or the occurrence of the primary event	Incomplete follow-up can occur in patients who fail to return to the clinic for follow-up, withdraw their consent or other reasons for discontinuation. These patients are censored in K–M survival analysis and excluded from per protocol analysis
Adequate clinical and parasitological response (ACPR)	Treatment success, defined as the absence of parasitaemia at the end of the study follow-up

The Kaplan–Meier (K–M) survival analysis is currently the approach recommended by the World Health Organization (WHO) for deriving antimalarial efficacy, where a competing risk event of a new infection is considered as a censored observation on the day of occurrence [2]. The complement of K–M estimate (1 minus K–M) is frequently reported in standalone efficacy studies as the WHO recommends replacing an existing treatment with an alternative regimen if the derived estimate of cumulative failure exceeds 10%. Several studies in clinical and statistical literature has shown that the 1 minus K–M approach provides an upwards biased estimate of the cumulative risk for the event of interest in the presence of competing risk events [1, 3–6]. In a re-analysis of an antimalarial efficacy trial from Uganda, it was demonstrated that the derived estimate of cumulative recrudescence using the 1 minus K–M approach can lead to a counter-intuitive scenario where the sum of the individual risk of recurrence for recrudescence and new infection is greater than for the composite endpoint of overall recurrence (see Figure 4 in [7]). An alternative approach for deriving failure estimate is to use the Cumulative Incidence Function (CIF) [8] (see Additional file 1: Section 1).

The presence of competing risk events can have further implications for comparative drug trials and regression modelling. First, comparative antimalarial trials utilize the widely used log-rank test to compare the efficacy between two drugs. An alternative approach, which compares the difference in cumulative risk between two groups by accounting for competing risk events, is the Gray’s *k*-sample test [9] (see Additional file 1: Section 2). Simulation studies have reported different performances of these two approaches depending on the underlying effect of the drug on the primary event of interest and on competing risk events [5, 10]. Second, in the presence of competing risk events, regression modelling can be carried out either using the Cox’s proportional hazard model or using the Fine and Gray’s model [11]. The former is based on modelling of the cause-specific hazard function whereas the latter is based on the modelling of sub-distribution hazard function. The differences between the cause-specific and sub-distribution hazard functions, and the underlying regression models are explained in Additional file 1: Sections 2, 3 and 4.

The application of competing risk survival analysis approach has gathered little attention in the antimalarial literature [7, 12]. This research aimed to address this research gap and there were three specific objectives:

i. To investigate the influence of competing risk events on the derived estimate of polymerase chain reaction (PCR) confirmed recrudescence in a stand-alone trial;
ii. To investigate the influence of competing risk events on the estimation of comparative efficacy between antimalarial drugs;
iii. To demonstrate regression modelling approaches in the presence of competing risk events.

## Methods

### Identification of studies for potential inclusion

The WorldWide Antimalarial Resistance Network (WWARN) repository contains a large collection of standardized data on antimalarial drugs [13]. Studies in the WWARN data repository were eligible for inclusion in the current analysis, if the data were from prospective clinical efficacy studies of uncomplicated *P. falciparum* (alone or mixed infections with *Plasmodium vivax*) in which patients were treated with one of the following fixed-dose regimens: artemether–lumefantrine (AL), dihydroartemisinin–piperaquine (DP), artesunate–amodiaquine (ASAQ), or artesunate–mefloquine (ASMQ). All studies also had to have applied molecular PCR genotyping to distinguish recrudescence from new infection. Studies on prophylactic use of antimalarials, severe malaria, pregnant women, patients with hyperparasitaemia, healthy volunteers, and travellers were excluded. Treatment outcomes were generated based on the definitions outlined in the WWARN Data Management and Statistical Analysis Plan [14].

### Statistical analyses

#### Derivation of cumulative failure estimates in standalone studies

The estimate of cumulative recrudescence at the end of study follow-up was derived using two methods: (i) the Kaplan–Meier method and (ii) the Cumulative Incidence Function (CIF) estimator (p. 255 of [15]). New infections and indeterminate outcomes were considered as censored at the time of their occurrence in the K–M approach whereas they were considered as different categories of competing events in the CIF approach. The difference in derived estimates of PCR confirmed recrudescence using these two different approaches was calculated and expressed in absolute scale. The effect of length of study follow-up, observed proportion of events and competing events per arm on the magnitude of the difference in the derived estimates between the two approaches was explored.

#### Impact of competing risk events in comparative efficacy studies

For comparative efficacy studies where the interest lies in establishing the difference between two drugs in terms of primary endpoint of interest (recrudescence), two different approaches were used: (i) the log-rank test to compare the equality of the K–M curves, and (ii) Gray’s *k*-sample test to compare the equality of the CIFs [16]. In the absence of competing risk events, the result of Gray’s *k*-sample test will be identical to that derived by the log-rank test [17]. In the log-rank test, new infections and indeterminate recurrence were considered as censored.

#### Regression models for recrudescence and new infection

In the presence of competing risk events, regression modelling can be carried out either on the cause-specific hazard function (using Cox proportional hazards model) or the sub-distribution hazard function (using Fine and Gray model) (see Additional file 1: Sections 3 and 4). A subset of data from a large multi-centre study (The 4ABC Trial [18]), which enrolled children aged less than 5 years in Africa was used to illustrate the two regression approaches in the presence of competing risk events.

The regression parameters of the Fine and Gray model were expressed as a sub-distribution hazard ratio (sdHR), and the output of the cause-specific Cox model as a cause-specific hazard ratio (csHR). Regression models were fitted without variable selection as the aim was to use the fitted model for risk prediction (rather than identification of putative factors) using the known confounders: age, baseline parasitaemia, and treatment regimen. The same set of covariates was used in models for recrudescence and new infection as recommended by Marubini and Valsechhi (p. 347) [19]. The fitted regression models were then used to estimate the predicted risk of recrudescence on day 28 (Additional file 1: Section 5).

#### Software

All the analyses were carried out using R software (Version 3.2.4) [20]. The log-rank test was carried out using the **survdiff** function in the *survival* package and Gray’s *k*-sample test was performed using the **cuminc** function in the *cmprsk* package.

## Results

### Characteristics of the studies and patients included

Individual patient data were available from 92 studies (31,507 patients) carried out in 169 trial sites with a total of 233 treatment arms (see Additional file 2 for details of the studies included). A total 186 arms (79.8%) were from Africa, 45 (19.3%) from Asia and 2 (0.85%) were from South America. The duration of follow-up was 28 days in 120 (51.5%) treatment arms, 42 days in 76 (32.6%) arms, and 63 days in 37 (15.9%) arms. Overall, 16,313 (51.9%) patients were treated with AL, 9064 (28.9%) with DP, 4782 (15.2%) with ASAQ and 1220 (3.9%) with ASMQ. Baseline characteristics of the patients are presented in Table 2.

**Table 2:** Baseline characteristics of patients included

Baseline characteristics	AL (1996–2014)	ASAQ (2002–2014)	ASMQ (2002–2013)	DP (2002–2013)	Total (1996–2014)
N	16,373	4850	1220	9064	31,507
Gender					
Female^a^	7394 (45.2%)	2253 (46.5%)	591 (48.4%)	3464 (38.2%)	13,702 (43.5%)
Age (years)^a^					
Mean age ± SD (years)	8.3 ± 10.66	7.0 ± 9.21	11.9 ± 13.37	13.5 ± 14.09	9.8 ± 11.94
< 1 year	924 (5.6%)	312 (6.4%)	65 (5.3%)	386 (4.3%)	1687 (5.4%)
1 to < 5 years	8629 (52.7%)	2895 (59.7%)	522 (42.8%)	3736 (41.2%)	15,782 (50.1%)
5 to < 12 years	3306 (20.2%)	773 (15.9%)	201 (16.5%)	1264 (13.9%)	5544 (17.6%)
≥ 12 years	3491 (21.3%)	862 (17.8%)	426 (34.9%)	3665 (40.4%)	8444 (26.8%)
Missing	23 (0.1%)	8 (0.2%)	6 (0.5%)	13 (0.1%)	50 (0.2%)
Continent^a^					
Africa	14,087 (86.0%)	4550 (93.8%)	810 (66.4%)	4862 (53.6%)	24,309 (77.2%)
Asia	2127 (13.0%)	300 (6.2%)	410 (33.7%)	3950 (43.6%)	6787 (21.5%)
South America	159 (1.0%)	0 (0.0%)	0 (0.0%)	252 (2.8%)	411 (1.3%)
Enrolment clinical parameters					
Mean body weight ± SD (kg)	20.9 ± 16.29	20.0 ± 16.02	23.7 ± 17.2	22.4 ± 17.55	21.2 ± 16.63
Mean haemoglobin ± SD (g/dL)	10.2 ± 2.21	9.6 ± 1.98	10.4 ± 2.26	10.2 ± 2.31	10.1 ± 2.22
Median parasitaemia [IQR] (/µL)	19,674 [5590–50,550]	21,730 [7353–53,439]	23,386 [5953–64,103]	14,320 [4178–43,337]	18,576 [5284–49,520]
Elevated temperature (> 37.5°)	64.3% [9705/15,101]	67.9% [3221/4747]	74.0% [743/1004]	60.2% [4200/6972]	64.2% [17,869/27,824]

*AL* artemether–lumefantrine, *ASAQ* artesunate–amodiaquine, *ASMQ* artesunate–mefloquine, *DP* dihydroartemisinin–piperaquine, *IQR* interquartile range, *SD* standard deviation, *N* number of patients

^a^Column percentages presented in parenthesis

### Study outcomes

**Fig. 2:**
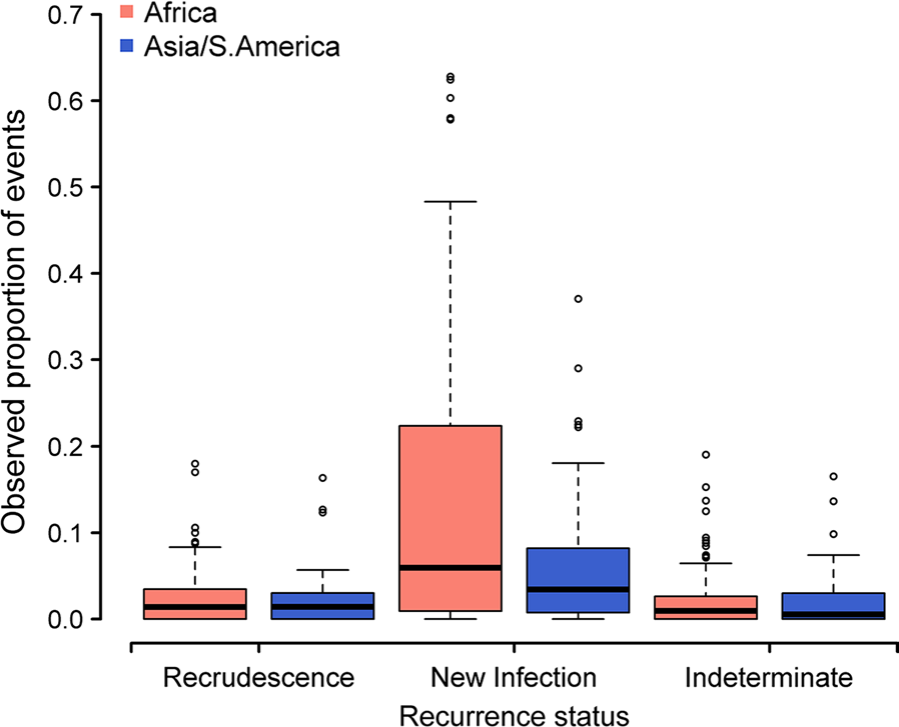
The observed proportion of recurrence events in the studies included. The distribution of observed proportion of recrudescences, new infections and indeterminate outcomes from 233 study arms included in the analysis

In Africa, a total of 4534 (18.8%) recurrent infections were documented, of which 553 (2.3%) were recrudescent infections; the proportion of recrudescent failures was 2.5% (356/14,027) for AL, 2.3% (112/4862) for DP, 1.6% (70/4482) for ASAQ and 1.9% (15/810) for ASMQ (Additional file 3: Section 1). In Asia, 8.5% (579/6787) of patients had recurrent infection, of which 126 (21.8%) were recrudescences. The proportion of patients with recrudescences in Asia was 2.7% (58/2127) for AL, 5.3% (16/300) for ASAQ, 3.2% (13/410) for ASMQ and 1.0% for DP (39/3950). In South America, there were 3 recrudescences (0.7%) and 4 new infections (1.0%). Of the 233 treatment arms, 83 (35.6%) arms reported no recrudescent infections, and 199 (85.4%) arms had at least one new infection observed. The observed proportion for the different event types are presented in Fig. 2.

### Standalone efficacy studies

#### Risk of *Plasmodium falciparum* recrudescence

**Fig. 3:**
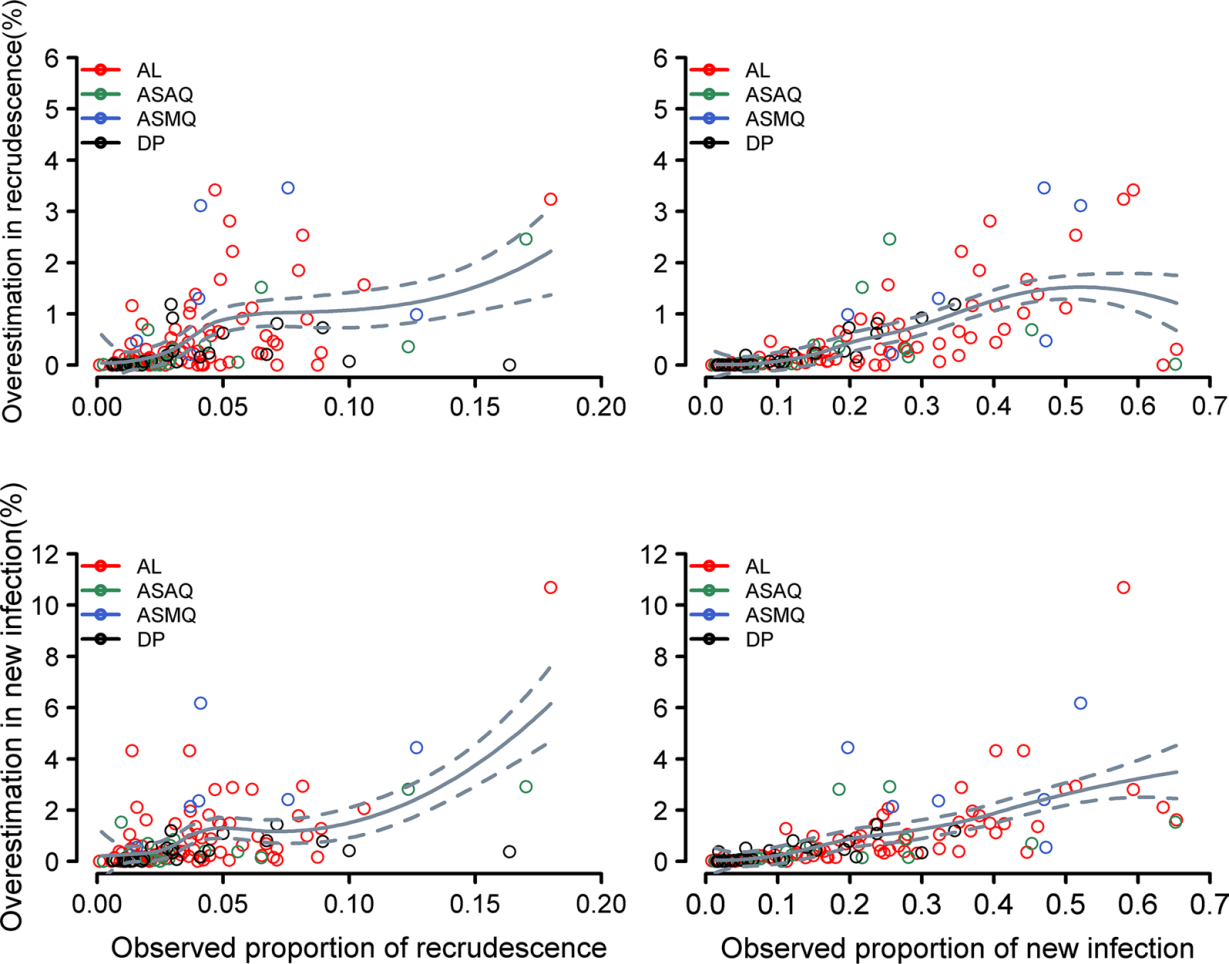
The overestimation of derived failure by 1 minus Kaplan–Meier method compared to the Cumulative Incidence Function. The overestimation $\left( {\hat{F}_{KM} \left( t \right) - \hat{F}_{CIF} \left( t \right)} \right)$ of cumulative recrudescence (top panel) and new infection (bottom panel) by using the Kaplan–Meier method plotted against observed proportion of recrudescence and proportion of new infections respectively. Estimates presented are at the end of the study follow-up. The grey trend line is a smoothed estimator obtained from local polynomial regression fitting, shown together with 95% confidence interval (outer dotted lines) for the overall data. *AL* artemether–lumefantrine, *ASAQ* artesunate–amodiaquine, *DP* dihydroartemisinin–piperaquine, *ASMQ* artesunate–mefloquine. Data are shown from the study arms where at least one recrudescence and at least one competing risk event were observed and from those arms where the number of patients at risk > 25 on the last day of the study follow-up

In 91 arms (39%), there was either absence of recurrence or only recrudescence or new infections were observed. In these arms, the failure estimates derived from both methods were identical. In the remaining 142 arms (61%), the 1 minus K–M method was associated with a marginal overestimation of the risk of PCR confirmed recrudescence compared to the CIF by a median of 0.04% [IQR: 0.00–0.27%; Range: 0.00–3.60%] (Fig. 3, upper panel). The degree of overestimation was progressively larger with increasing study follow-up duration; the median overestimation being 0.006% [IQR: 0.00–0.07%; Range: 0.00–2.54%] on day 28, 0.15% [IQR: 0.00–0.57%; Range: 0.00–3.23%] on day 42, and 0.56% [IQR: 0.11–1.12%; Range: 0.00–3.60%] on day 63 (Table 3). The magnitude of overestimation also correlated with the observed proportion of new infections [Pearson’s correlation coefficient: 0.43; 95% CI 0.35–0.54] and the observed proportion of recrudescences [Pearson’s correlation coefficient: 0.38, 95% CI 0.30–0.46] (Fig. 3, Table 3).

**Table 3:** Absolute overestimation (%) in cumulative recrudescence estimates using K–M analysis compared to Cumulative Incidence Function

	*N*	Absolute overestimation^a^	Observed proportion of recrudescence^b^	Observed proportion of competing risk events^b^
Day 28				
AL	88	0.02% [IQR: 0.00–0.12%; Range: 0.00–2.54%]	2.1% [0.1–16.0%]	9.8% [0.0–51.4%]
ASAQ	21	0.03% [IQR: 0.00–0.10%; Range: 0.00–0.98%]	2.5% [0.3–12.8%]	10.2% [0.0–45.4%]
ASMQ	8	0.00% [IQR: 0.00–0.03%; Range: 0.00–0.08%]	1.9% [0.8–7.0%]	11.0% [6.3–14.0%]
DP	30	0.00% [IQR: 0.00–0.00%; Range: 0.00–0.16%]	1.3% [0.4–16.4%]	2.7% [0.0–36.8%]
Day 42				
AL	48	0.25% [IQR: 0.03–0.65%; Range: 0.00–3.24%]	3.7% [0.6–18.0%]	21.6% [0.0–65.4%]
ASAQ	9	0.23% [IQR: 0.02–0.69%; Range: 0.00–2.24%]	3.3% [1.0–17.0%]	18.4% [2.3–65.2%]
ASMQ	8	0.25% [IQR: 0.12–0.50%; Range: 0.00–1.03%]	3.7% [1.4–11.3%]	25.3% [1.0–43.3%]
DP	21	0.00% [IQR: 0.00–0.19%; Range: 0.00–0.73%]	1.9% [0.8–16.4%]	7.1% [0.0–34.6%]
Day 63				
AL	9	0.96% [IQR: 0.65–1.38%; Range: 0.012–3.42%]	3.7% [1.4–10.6%]	7.1% [0.0–34.6%]
ASAQ	2	0.18% [IQR: 0.09–0.27%; Range: 0.00–0.36%]	12.3%	18.5%
ASMQ	7	0.98% [IQR: 0.38–2.20%; Range: 0.21–3.59%]	4.1% [1.6–12.7%]	4.1% [1.6–12.7%]
DP	8	0.14% [IQR: 0.01–0.38%; Range: 0.00–0.89%]	2.2% [1.6–12.7%]	7.1% [3.4–30.0%]

*N* number of study sites with study sample size ≥ 25, *AL* artemether–lumefantrine, *ASAQ* artesunate–amodaiquine, *DP* dihydroartemisinin–piperaquine, *ASMQ* artesunate–mefloquine, *IQR* interquartile range

^a^Values are median [Interquartile Range (IQR); Range] for $\hat{F}_{KM} \left( t \right) - \hat{F}_{CIF} \left( t \right)$

^b^Values are median [Range]

The maximum overestimation was 3.6%, which occurred in an artesunate–mefloquine arm in Balonghin site in Burkina Faso (n = 66), an area of high transmission [21]. In this arm, the day 63 failure estimate derived using the K–M method was 20.0% [95% CI 0.0–55.1] and the corresponding CIF estimate was 16.4% [95% CI 0.0–48.6]. In 9.0% (21/233) of the treatment arms, the overestimation was greater than 1%, in 4.3% (10/233) this was greater than 2%, and in 2.6% (6/233) the difference was greater than 3%. All of the 21 study sites where the overestimation exceeded 1% were from Africa except one from Papua New Guinea, where 51% of patients with parasite recurrence were due to to *P. vivax*.

#### Study sites where estimate of PCR-confirmed recrudescence exceeded 10% using complement of K–M

In three (1.3%) study arms, the estimated cumulative risk of recrudescence exceeded 10% (the WHO threshold for withdrawing first line therapy) based on the K–M method, but the CIF estimates were all less than 10% (Table 4). Similarly, in 9 (3.9%) study arms, the estimated failures were greater than 5% (the WHO threshold required to meet for introducing a regimen as a first line therapy) using the K–M method, all of which were less than 5% using the CIF.

**Table 4:** Study sites where cumulative failure estimates exceeded 10% and 5% using K–M approach

Study (site)	N	Number of events (RC/NI/IND)	Day	Drug	$\hat{F}_{KM} \left( t \right)$ estimate of recrudescence [95% confidence interval]	$\hat{F}_{CIF} \left( t \right)$ estimate of recrudescence [95% confidence interval]
10% threshold^a^						
Nikiema-2010 (Burkina Faso, Gourcy)^b^	144	12/28/3	28	AL	10.3 [4.8–15.8]	9.4 [4.3–14.5]
The 4ABC Trial (Burkina Faso, Nanoro) [18]	294	24/142/9	28	AL	11.1 [6.8–15.4]	8.6 [5.3–11.8]
Sirima-2015 (Burkina Faso, Balonghin) [21]	66	31/5/0	63	ASMQ	11.5 [7.2–15.9]	8.1 [1.2–14.9]
5% threshold^a^						
Yeka-2008 (Uganda, Kanungu) [45]	199	9/49/6	42	AL	5.2 [1.9–8.5]	4.6 [1.7–7.6]
Sirima-2015 (Tanzania, Korogwe) [21]	27	1/3/2	63	AL	5.0 [0.0–14.6]	4.3 [0.0–12.9]
Schramm-2013 (Liberia, Nimba) [46]	145	7/45/6	42	AL	5.6 [1.5–9.7]	5.0 [1.4–8.6]
Agrawal-2013 (Kenya, Siaya) [47]	136	5/48/12	42	AL	5.2 [0.5–10.0]	4.2 [0.6–7.8]
Karunajeewa-2008 (PNG, Madang) [48]	54	2/20/0	42	AL	5.9 [0.0–14.3]	4.7 [0.0–11.3]
Sirima-2015 (Kenya, Kisumu) [21]	99	4/23/9	63	ASMQ	6.1 [0.1–12.0]	4.8 [0.2–9.4]
Sirima-2015 (Burkina Faso, Balonghin) [21]	128	5/58/1	63	AL	5.4 [0.7–10.2]	4.0 [0.6–7.5]
Bukirwa-2006 (Uganda, Tororo) [49]	204	10/89/2	28	AL	6.6 [2.6–10.7]	4.9 [2.0–8.0]
Sirima-2015 (Kenya, Ahero) [21]	73	3/28/10	63	ASMQ	7.8 [0.0–16.7]	4.7[0.0–10.0]

*N* study sample size, *RC* recrudescence, *NI* new infection, *IND* indeterminate outcomes, $\hat{F}_{KM} \left( t \right)$ cumulative failure estimates derived using 1 minus Kaplan–Meier method, $\hat{F}_{CIF} \left( t \right)$ cumulative failure estimates derived using Cumulative Incidence Function, *AL* artemether–lumefantrine, *ASMQ* artesunate–mefloquine

^a^ The 10% threshold is used by the WHO for determining whether the current regimen should be continued to be used as a first line therapy and 5% threshold is used for introducing a new regimen as a first line treatment [50]

^b^ Unpublished study

#### Risk of *Plasmodium falciparum* new infection

The median overestimation of the cumulative risk of new *P. falciparum* infections during the follow-up period using the K–M method (which considered recrudescences as censored) compared to the Cumulative Incidence Function was 0.39% [IQR: 0.08–1.10%; Range: 0.00–10.60%]. The overestimation progressively increased with the follow-up duration, which was 0.10% on day 28, 0.67% on day 42, and 1.40% on day 63 (Fig. 3; lower panels). The overestimation increased with increasing proportion of patients with new infections and recrudescences observed in a study. The maximum overestimation was 10.6%, observed in a study with artemether–lumefantrine (n = 50) carried out in Tanzania. In this study arm, there were 9 (18%) recrudescences, and 29 (58%) new infections. Using the 1 minus K–M method, the risk of new infection on day 42 was 68.6% [95% CI 54.3–83.1] and this was 58.0% [95% CI 44.2–71.7] using the CIF method.

### Comparative efficacy studies

The results of the comparative efficacy studies are presented in Additional file 3: Section 2. There was no difference in the overall conclusion derived (at 5% level of significance) using the two approaches for testing for equality between the drug regimens using the log-rank test and Gray’s *k*-sample test.

### Regression models for time to recrudescence

Data from 810 children enrolled in Burkina Faso (a subset of the The 4ABC Trial [18]) treated with artemether–lumefantrine (AL) (n = 294), artesunate–amodiaquine (ASAQ) (n = 295) and dihydroartemisinin–piperaquine (DP) (n = 221) were used to illustrate the regression modelling approaches in the presence of competing risk events. The observed proportion of patients with recrudescences were 3.2% in the DP arm, 8.2% in the AL arm, and 3.1% in the ASAQ arm, while the respective proportion for new infections were 10.0%, 48.3%, and 25.1% (Table 5). In a multivariable model for recrudescence (which included age, baseline parasitaemia and treatment regimen), age and baseline parasitaemia did not reach conventional statistical significance for recrudescence, neither in the cause-specific hazard model nor in the sub-distribution hazard model (*P*-value > 0.05). Treatment with AL (relative to DP) was associated with increased cause-specific hazard and increased sub-distribution hazard of recrudescence (csHR = 4.02 [95% CI 1.72–9.43]; sdHR = 2.85 [95% CI 1.24–6.57]).

**Table 5:** Regression models for recrudescence and new infection using data from Burkina Faso (n = 810) [18]

	Observed proportion of events	Cause-specific Cox proportional hazard model		Fine and Gray’s sub-distribution hazard model	
		Cause-specific HR [95% confidence interval]	*P*-value	Sub-distribution HR [95% confidence interval]	*P*-value
Model for recrudescence					
Age (/yearly increase)	4.9% (40/810)	0.99 [0.77–1.29]	0.965	1.00 [0.80–1.25]	0.990
Baseline parasitaemia (/µL) (tenfold increase)	4.9% (40/810)	1.67 [0.88–3.16]	0.114	1.44 [0.84–2.46]	0.190
Treatment (ref = DP)	3.2% (7/221)				
AL	8.2% (24/294)	4.02 [1.72–9.43]	0.001	2.85 [1.24–6.57]	0.014
ASAQ	3.1% (9/295)	1.12 [0.42–2.99]	0.829	1.01 [0.38–2.69]	0.980
Model for new infection					
Age (/yearly increase)	29.4% (238/810)	1.08[0.98–1.21]	0.121	1.08[0.99–1.19]	0.093
Baseline parasitaemia (/µL) (tenfold increase)	29.4% (238/810)	1.52[1.18–1.97]	0.001	1.40[1.10–1.78]	0.006
Treatment (ref = DP)	10.0% (22/221)				
AL	48.3% (142/294)	8.05[5.12–12.67]	< 0.001	6.51[4.27–9.94]	< 0.001
ASAQ	25.1% (74/295)	3.03[1.88–4.88]	< 0.001	2.83[1.80–4.44]	< 0.001

*AL* artemether–lumefantrine, *DP* dihydroartemisinin–piperaquine, *ASAQ* artesunate–amodiaquine

**Fig. 4:**
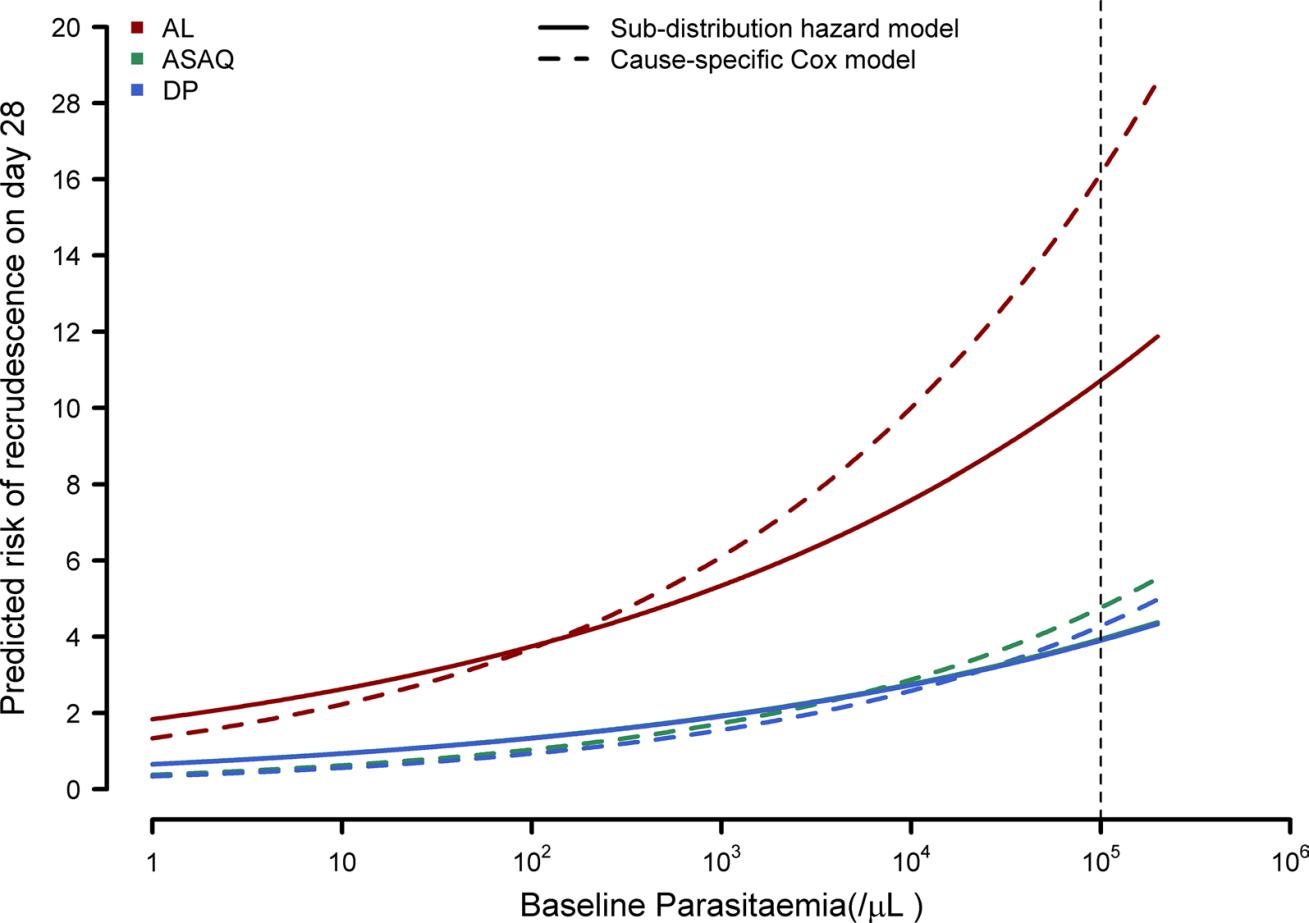
Predicted risk of recrudescence from cause-specific Cox model and sub-distribution hazard model. The graph was generated using the regression coefficients presented in Table 5 and the estimate of baseline hazard obtained from the respective sub-distribution and cause-specific hazard model (for a 3 year old child). The cumulative baseline sub-distribution hazard on day 28 from Fine and Gray’s model was 0.006; the cumulative baseline hazard on day 28 from the cause-specific Cox model was 0.003. The vertical dotted line represents the parasitaemia of 100,000/µL for the child described in the main text. On day 28, the predicted risk of recrudescence for this patient was 16.15%, 4.76% and 4.28% using the cause-specific Cox model. The corresponding figures were 10.72%, 4.76% and 4.28% with the Fine and Gray’s sub-distribution hazard model. *AL* artemether–lumefantrine, *ASAQ* artesunate–amodiaquine, *DP* dihydroartemisinin–piperaquine

The regression models presented in Table 5 were used for predicting the risk of recrudescence on day 28 for a patient aged 3 years old with an initial parasite load of 100,000/µL. For the DP regimen, the predicted risk for a patient with this covariate profile was 4.3% using the cause-specific Cox proportional hazard model, and 3.9% using the sub-distribution hazard model (Fig. 4). For the AL regimen, the predicted risks were 16.2% and 10.7% using the cause-specific and sub-distribution hazard models respectively. For ASAQ, the estimates of predicted risks were 4.8% using the cause-specific Cox model and 3.9% using the Fine and Gray’s sub-distribution hazard model.

## Discussion

Recent reviews have shown that the majority of studies published in medical journals are susceptible to competing risk biases [6, 16, 22]; a concept hitherto overlooked in malaria literature. This re-analysis of individual patient data of 233 treatment arms from 92 clinical efficacy studies conducted in Asia, Africa, and South America revealed that just over a third (83/233) of the treatment arms had an observed proportion of new infection greater than 10%, a threshold considered to make studies vulnerable to competing risk bias [23]. This suggests that competing risk events are the rule rather than the exception in antimalarial trials.

This analysis allowed the exploration of the degree to which the derived estimate of failure was affected by ignoring the competing risk events in analysis of antimalarial efficacy trials. The K–M analysis which censored new infections was associated with a marginal absolute overestimation of the cumulative risk of recrudescence. In 9% (21/233) of the study arms the overestimation was greater than 1%, in 4.3% (10/233) the difference was greater than 2%, and in 2.6% (6/233) of the arms the difference was greater than 3%. All but one of the 21 study sites where the difference exceeded 1% were from Africa (areas of intense malaria transmission), the exception being a study from Papua New Guinea where a very high proportion of patients experienced recurrent parasitaemia due to *P. vivax*. The degree to which K–M overestimated failure in a study arm was correlated with the proportion of patients experiencing new infection or recrudescence, and the follow-up duration; these findings are consistent with the literature [1].

The current WHO guidelines recommend that when the estimates of recrudescence at the end of the follow-up exceed 10%, a series of detailed clinical, pharmacological and in vitro investigations should be undertaken to examine the possibility of parasite drug resistance. If resistance is confirmed, then treatment policy should be revised to a more effective regimen [2]. In three study arms, the estimated failure was greater than 10% (the WHO threshold for withdrawing antimalarials) when the K–M method was used, but remained below 10% when using a competing risk survival analysis method with the 95% confidence interval for the two estimates overlapping and the estimated 95% confidence interval included this threshold (Table 4). However, if the clinical decision-making was based solely on the point-estimates, then this highlights that ignoring competing risk events can result in potentially misleading conclusions being drawn from an efficacy trial, especially when the derived estimates are at the cusp of these thresholds.

The effect of competing risk events in comparative settings was then evaluated, as the partner components of the ACT are eliminated at different rates resulting in a differential fraction of new infections observed. For example, lumefantrine has a much shorter terminal elimination half-life compared to piperaquine [24]. The underlying drug pharmacokinetics will result in a lower observed proportion of new infections following DP administration compared to the AL regimen, especially in areas of intense malaria transmission [25]. This highlights the importance of taking the proportion of competing risk events into consideration when comparing drug regimens with different pharmacological properties. In order to explore whether these pharmacological differences affected the comparative analyses of these two drugs, the equality of the survival curves was compared using the log-rank test and Gray’s *k*-sample test using data from 27 comparative studies. There were no apparent differences in the derived conclusions using these two approaches as there were very few observed recrudescences in each of the study arms (Additional file 3: Section 2).

Finally, two different approaches to regression modelling in the presence of competing risk events were presented using data from Burkina Faso: the regression model on cause-specific hazard and on the sub-distribution hazard. The estimates of the sub-distribution hazard ratio (sdHR) were somewhat attenuated and closer to the null value compared to the cause-specific hazard ratio (csHR) (Table 5). Although the relative risk measures (cause-specific hazard ratio and sub-distribution hazard ratio) obtained from these two regression models were similar (Table 5), they are not directly comparable as they have a different interpretation [26, 27]. In the illustrated example, the csHR of 1.67 for baseline parasitaemia implies that every tenfold rise in parasite load was associated with a 1.67-fold higher risk of recrudescence, among patients who had not experienced any recurrence yet by the end of the follow-up. The sdHR of 1.44 (higher than 1) means that the cumulative incidence of recrudescence increases with every tenfold increase in parasite density and the interpretation of the numeric value of 1.44 is not straight forward [26]. This is because subjects who have experienced new infections are still maintained in the risk-set when computing a sub-distribution hazard, even though they are no longer at risk of experiencing recrudescence (Additional file 1: Section 3).

In the presence of competing risk events, researchers are faced with a choice of methods, and this has gathered considerable attention in medical and statistical literature [10, 17, 28–30]. In comparative studies, the log-rank test is considered appropriate when the research interest is in understanding the biological mechanism of how a treatment affects recrudescence (hazard rate). If the research interest is to answer if subjects receiving a particular drug are more likely to experience recrudescence at the end of the study follow-up, the comparison of CIF through Gray’s *k*-sample test is considered appropriate [1, 31, 32]. Many authors advocate presenting results of both these approaches to provide a complete biological understanding of the treatment on the different endpoints [1, 33]. For regression models, if the aim is to estimate probability and provide evidence to inform medical decision-making, the use of sub-distributional hazard model has been advocated as the method of choice [27, 34, 35], and if the aim is to explore the underlying biological effects of a covariate on the outcome, then a regression model on the cause-specific hazard has been preferred [1, 26, 27, 34].

This analysis has a number of limitations. It was assumed that the outcome of molecular genotyping reflects the true treatment outcomes. The current approach to parasite genotyping applies a conservative approach which overestimates the recrudescence particularly in areas of intense malaria transmission [36, 37]. In areas of very high transmission, such as Uganda, parasite infections are frequently polyclonal and as many as 45% of the recrudescences could be misclassified as new infections [38]. Further difficulties arise when the subsequent recrudescence is due to a minority clone which was undetected at baseline, thus leading to misclassified outcomes. This necessitates incorporating the uncertainity around the outcome classification, such as by using a Bayesian approach for classification of late treatment failures [39]. Indeterminate outcomes were censored in K–M analysis and considered as an extra category of competing risk event when generating CIF. An indeterminate outcome can only be considered as a competing risk event if the new infection and recrudescence coincide. However, an indeterminate outcome arising for other reasons, such as missing pairwise samples, or failure to amplify the parasite DNA cannot be considered as competing events in sensu stricto. In such a situation, considering them as an extra category for CIF analysis might have introduced bias. The efficacy of ACT in uncomplicated *falciparum* malaria remains high, with treatment failure reported in less than 5% of patients in the vast majority of ACT studies included in this analysis. Hence, the data used in this re-analysis did not allow the investigation of what would happen when the antimalarial efficacy declines. In order to quantify the magnitude of the potential biases in situations of falling antimalarial efficacy (now observed for dihydroartemisinin–piperaquine in Cambodia and Vietnam [40–42]), simulation studies were conducted and reported elsewhere [43]. This study did not explore the scenario of multiple-failure time (multivariate survival data), in which each patient can experience multiple events during follow-up. This scenario is of low relevance for *falciparum* malaria, but multiple events frequently occur in trials of *vivax* malaria, in which a patient may experience multiple relapses due to reactivation of hypnozoites from the liver.

Finally, it is difficult to disentangle whether a new infection is a truly competing endpoint in a biological sense. In a situation where the parasite causing the initial infection is *subdued* at low density and the newly emerging infection has higher density than the *subdued* original infection, then on a strong assumption of identical parasite multiplication rate for both infections, this constitutes a situation where the occurrence of new infection precludes recrudescence (Fig. 1a). This assumption of identical parasite multiplication rates might be plausible for rapidly eliminated drugs. For slowly eliminated drugs, the parasite growth rate for recrudescence and a new infection are likely to be different. Similarly, a recrudescent infection where the parasite numbers never go below those encountered at the beginning of a new infection cannot be pre-empted and the occurrence of recrudescence will not be affected (Fig. 1b). The only exception is if the new infection is more resistant than the primary infection. When the initial infection is completely eliminated after exposure to antimalarials, the host is no longer at a risk of subsequent recrudescence and competing risk situation does not exist (Fig. 1c). In reality it is impossible to disentangle the underlying in vivo parasitological circumstances from this dataset. Thus, new infections can be considered as a “*situational competing risk event*” which is primarily dependent on the inoculum density, fitness, efficiency of a newly emergent infection and of the existing recrudescent parasites, and the host immunity.

## Conclusions

Censoring competing events in the Kaplan–Meier analysis led to an overestimation of the risk of recrudescence, which was of marginal clinical importance in the data included in this analysis. In the areas of high transmission where a large proportion of recurrences are attributable to new infections, the use of CIF provides an alternative approach for the derivation of failure estimates for anti-malarial treatments.

## Additional files

**Additional file 1.** Definitions.

**Additional file 2.** List of studies used.

**Additional file 3** Additional results.

## Abbreviations

$\hat{F}_{KM} \left( t \right)$: The complement of Kaplan–Meier estimate [1 − $\hat{S}_{KM} \left( t \right)$]
$\hat{S}_{KM} \left( t \right)$: Kaplan–Meier estimates of drug efficacy at time *t*
CIF: Cumulative Incidence Function
csHR: Cause-specific hazard ratio
K–M: Kaplan–Meier estimate
NI: New infection
RC: Recrudescence
sdHR: Sub-distribution hazard ratio
WHO: World Health Organization

## Availability of data and materials

The database(s) supporting the conclusions of this article are available from the WorldWide Antimalarial Resistance Network (WWARN) (http://www.wwarn.org/), a controlled open access repository of antimalarial studies on reasonable request.
