# Supplementary material for: Competing risk events in antimalarial drug trials in uncomplicated Plasmodium falciparum malaria: a WorldWide Antimalarial Resistance Network individual participant data meta-analysis
Source: Malar J. 2019 Jul 5;18:225. doi: 10.1186/s12936-019-2837-4 (PMC6612160; doi:10.1186/s12936-019-2837-4)
Supplement: Supplementary file 1 — Additional file 1. Definitions. [file 12936_2019_2837_MOESM1_ESM.docx]

**Additional file 1:**

**Competing risk events in antimalarial drug trials in uncomplicated *Plasmodium falciparum* malaria: A WorldWide Antimalarial Resistance Network Individual Participant Data Meta-Analysis**

The WorldWide Antimalarial Resistance Network Methodology Study Group ^1^

^1^WorldWide Antimalarial Resistance Network (WWARN), Centre for Tropical Medicine and Global Health, Nuffield Department of Clinical Medicine, University of Oxford, Oxford, UK

Correspondences to

prabin.dahal@wwarn.org

[kasia.stepniewska@wwarn.org](mailto:kasia.stepniewska@wwarn.org)

1. **Kaplan-Meier estimates and Cumulative Incidence function**

The estimate of cumulative incidence of recrudescence at time *t* using the Kaplan-Meier (K-M) method is obtained as:

$$\hat{F}_{KM}\left( t \right)= 1-\hat{S}_{KM}\left( t \right)=1-\left( \prod_{\tau\leq t} \frac{n_{\tau}-d_{\tau}}{n_{\tau}} \right)$$

where $d_{\tau}$is the number of recrudescences at time $\tau$, $n_{\tau}$ is the total number of patients at risk of experiencing recrudescence just before time $\tau$ (the risk-set). The **Cumulative Incidence Function (CIF)** estimator proposed by Kalbfleisch and Prentice (p. 255 of [1]) provides the probability of failure by appropriately accounting for competing risk events. The CIF at time t for an event *i* is given by:

$${\hat{F}_{CIF}}_{i}(t)=\sum_{\tau\leq t} \frac{d_{i\tau}}{n_{\tau}}.\hat{S_{KM}}(\tau-1)$$

where $d_{i\tau}$ is the number of events of type $i$ that occur at time$\tau$, and $n_{\tau}$ is the number of patients at risk at time $\tau$, and $\hat{S}_{KM}(\tau-1)$ is the K-M estimator of the survival probability of being free of any event type prior to time $\tau$. Mathematically, it has been shown that $\hat{F_{KM}}\left( t \right)$≥${\hat{F_{CIF}}}_{i}(t$) and thus Kaplan-Meier approach for deriving estimate of drug failure leads to an overestimation in failure [2, 3].

1. **Comparing treatment regimens**
   1. ***Rates versus risks***

In survival analysis, inferences can be drawn on either of the two epidemiological quantities of fundamental interest: **rates or risks** [4]. The rates are characterised by the **hazard function**, which is an instantaneous probability of observing the event of interest at a given time-point *t* among those individuals who are still in the risk-set. **Risk** is a cumulative measure and is defined as the cumulative probability of the event of interest accrued over the time period under consideration. In standard survival analysis (where competing risk events are assumed not to occur), there is a simple one-to-one correspondence between the hazard function (rate) and the cumulative failure function (risk). This means that if the effect of an exposure variable is to increase the instantaneous hazard rate of the event of interest *k*, then its effect on the cumulative risk of the event *k* is also the same.

- 1. ***Log-rank test and Gray’s k-sample test***

Comparative antimalarial studies uses log-rank test for establishing differences between two drugs at the end of the study follow-up. The log-rank test is essentially the comparison of the underlying hazard rates between two groups as noted by Bajorunaite and Klein (2007) [5] and Dignam and Kocherginsky (2008) [6]. The one-to-one correspondence between rate and risk means that the log-rank test for comparing the equality of hazard rates is also equivalent to comparing the equality of survivals. A corollary of this statement is that any inference drawn upon the hazard function holds equivalently true for the survival function and the cumulative incidence. However, in the presence of competing risk events, the one-to-one relationship between the (**cause-specific**) **hazard function** (explained in section to follow) and the corresponding cumulative failure function no longer holds true [7], and the effect of the exposure variable on the hazard rate for event *k* may not coincide with its effect on the risk of event *k*.

An alternative approach, which compares the difference between two exposure groups accounting for the presence of competing risk events, is **Gray’s *k*-sample test** [8]. This is the usual log-rank test where the cause-specific hazard function is replaced by the hazard of the sub-distribution (as explained in the section above) [9]. In the absence of competing risk events, the result of a Gray’s *k*-sample test will be identical to that derived from a log-rank test [6].

1. **Cause specific hazard (CSH) and sub-distribution hazard (SDH)**

***The cause-specific hazard***: The cause-specific hazard defines the instantaneous risk of an event occurring per unit time for the specific cause of the event, among subjects without any prior events. This is the probability of failure due to cause *k* (for example, recrudescence) at time *t*, given that no failures of any kind (neither recrudescence nor new infection) have occurred thus far. The cause-specific hazard at a given time point for an event type can be computed by dividing the number of individuals experiencing that event type by the total number of individuals who were in the risk-set immediately prior to the time *t*. Individuals are removed from the risk-set if they experience either of the two events or those who are lost to follow-up.

***The sub-distribution hazard*:** The sub-distribution hazard function, introduced by Fine and Gray [10], is the probability of observing an event *k* in the next time interval, given that no cause *k* failures (neither recrudescence nor new infection) have occurred thus far. The sub-distribution hazard at a given time-point for an event type can be computed by dividing the number of individuals experiencing the event type (e.g. recrudescence) by the total number individuals who were in the risk-set immediately prior to the time *t*, while still maintaining individuals who experienced competing events in the risk-set. This is the main difference between cause-specific hazard and the sub-distribution hazard approach. Maintaining subjects with a new infection in the risk-set allows them to act as a place holder that represents the proportion of the risk-set that can never experience the primary outcome [11]. Like with the cause-specific hazard function, those who are lost to follow-up are removed from the risk-set.

1. **Regression models for recrudescence and new infections**

In the presence of competing risk events, regression modelling can be carried out either on the cause-specific hazard function or the sub-distribution hazard function.

Let there be two events$(j)$, where $j=1$ indicates recrudescence (the primary event of interest) and $j=2$ indicates a new infection (a competing risk event). The cause-specific hazard model for an event $j$ can be fitted by using the Cox proportional hazard model and censoring the competing risk event. This is referred to as the cause-specific Cox proportional hazard model. This is expressed as:

$h_{j}\left( t|\boldsymbol{x} \right)=h_{0j}\left( t \right).exp(\boldsymbol{x}^{'}\beta_{j})$ $j=1,2$

where $\beta_{j}$ is the vector of the coefficients of the set of explanatory variables $\boldsymbol{x}$, and $h_{0j}\left( t \right)$ is the cause-specific baseline hazard function for cause $j$ at time$t$. The value $exp(\beta_{j})$ is interpreted as the cause-specific hazard ratio (csHR) for the $j^{th}$ event corresponding to a unit change in the value of the corresponding covariate. This model assumes that the hazard remains proportional, which means the relative hazard for two groups remains constant throughout the time-period under study. The cause-specific Cox model can be equivalently written in terms of the baseline survival function ($S_{0}(t)$) for event type $j$as:

$S_{j}\left( t | \boldsymbol{x} \right)={S_{0j}(t)}^{exp(\boldsymbol{x}^{'}\beta_{j})}$ $j=1,2$

The cause-specific Cox model estimates the relative effect of the covariates on the cause-specific hazard function. This denotes the relative change in the *instantaneous hazard rate* of occurrence of the event of interest in subjects who are currently event-free. In the absence of competing risk events, a one-to-one correspondence is maintained between the rate and risk, thus there is a direct correspondence between the effect of the covariate on the hazard of the outcome and the effect of the covariate on the cumulative incidence (risk) of the outcome. However, in the presence of competing risk events, the association of a covariate with an increased (or decreased) cause-specific hazard rate doesn’t necessarily translate to an increased (or decreased) incidence of the event of interest [7]. This is because one must account for the effect of a covariate on the cause-specific hazard function of each of the competing risk events in order to fully determine the effect of that covariate on the incidence of the event of interest [12]. Hence, cause-specific regression modelling for the event of interest $j$ is limited when the interest is in drawing inference on the cumulative incidence of that event [12].

In the competing risk framework, the influence of covariates on the cumulative incidence of the event of interest can be explored by the sub-distribution hazard model (also called the CIF regression model) proposed by Fine and Gray [10]. The proportional sub-distribution hazard model can be written in a similar way to the Cox proportional hazard model by replacing the cause-specific baseline hazard with the baseline sub-distribution hazard:

$\lambda_{j}\left( t|\boldsymbol{x} \right)=\lambda_{0j}\left( t \right).exp(\boldsymbol{x}^{'}\varphi_{j})$ $j=1,2$

where $\varphi_{j}$ is the vector of the coefficients of the set of explanatory variables $\boldsymbol{x}$, and $\lambda_{0j}\left( t \right)$ is the baseline sub-distribution hazard function for cause $j$ at time$t$. The value $exp(\varphi_{j})$ is interpreted as the sub-distribution hazard ratio (sdHR) for a given covariate on the $j^{th}$ event. This model makes an explicit link between the sub-distribution hazard function and allows estimation of the effect of a covariate on the incidence of the event of interest. Following Austin and Fine (2017) [12], Fine and Gray’s sub-distribution hazard model can also be written in terms of cumulative incidence function (CIF) as:

$CIF_{j}\left( t|\boldsymbol{x} \right)={1-\left( 1-CIF_{0j}\left( t \right) \right)}^{\exp\left( \boldsymbol{x}^{'}\varphi_{j} \right)}$ $j=1,2$

where $CIF_{0j}\left( t \right)$ is the baseline CIF for an event $j$.

1. **Estimation of predicted values from Cox regression model and Fine and Gray model**

For a set of p covariates ($x_{i}$) at time *t*, the predicted risk of recrudescence from Fine and Gray’s sub-distribution hazard model ($\hat{F}_{FG})$ is obtained using:

$$\hat{F_{FG}}\left( t | x_{i} \right)=1-\exp\left( -\exp\left( \sum_{k=1}^{p} \beta_{k}x_{ik} \right).\int_{0}^{t} \bar{h_{1,0}}\left( s \right)ds \right)$$

where $\int_{0}^{t} \bar{h_{1,0}}\left( s \right)ds$is the cumulative baseline sub-distribution hazard at time *t* and $\beta_{k}$ is the regression coefficient for the *k*th covariate.

The predicted risk from Cox proportional hazard model ($\hat{F}_{Cox})$ can be obtained using:

$$\hat{F_{Cox}}\left( t | x_{i} \right)=1-\left( {\hat{S}_{0}\left( t \right)}^{exp(\sum_{k=1}^{p} \beta_{k}x_{ik}))} \right)$$

where $\hat{S_{0}}\left( t \right)$is the baseline survival obtained using $\hat{S_{0}}\left( t \right)=exp(-\hat{H_{0}}\left( t \right))$, and $\hat{H_{0}}\left( t \right)$ is the cumulative baseline hazard at time *t* obtained from the Cox model.

1. **References**

1. Kalbfleisch JD, Prentice RL. Competing Risks and Multistate Models. In: The Statistical Analysis of Failure Time Data. 2nd edition. New York, USA: John Wiley and Sons Inc; 2002. p. 247–77.

2. Gooley TA, Leisenring W, Crowley J, Storer BE. Estimation of failure probabilities in the presence of competing risks: New representations of old estimators. Stat Med. 1999;18:695–706.

3. Pintilie M. Analysing and interpreting competing risk data. Stat Med. 2007;26:1360–7.

4. Wolkewitz M, Cooper BS, Bonten MJM, Barnett AG, Schumacher M. Interpreting and comparing risks in the presence of competing events. BMJ. 2014;349:g5060.

5. Bajorunaite R, Klein JP. Comparison of failure probabilities in the presence of competing risks. J Stat Comput Simul. 2008;78:951–66.

6. Dignam JJ, Kocherginsky MN. Choice and interpretation of statistical tests used when competing risks are present. J Clin Oncol. 2008;26:4027–34.

7. Andersen PK, Geskus RB, De witte T, Putter H. Competing risks in epidemiology: Possibilities and pitfalls. Int J Epidemiol. 2012;41:861–70.

8. Gray RJ. A Class of K-Sample Tests for Comparing the Cumulative Incidence of a Competing Risk. Ann Stat. 1988;16:1141–54.

9. Klein JP. Competing risks. Wiley Interdisciplinary Reviews: Computational Statistics. 2010;2:333–9.

10. Fine JP, Gray RJ. A Proportional Hazards Model for the Subdistribution of a Competing Risk. J Am Stat Assoc. 1999;94:196–509.

11. Lau B, Cole SR, Gange SJ. Competing risk regression models for epidemiologic data. Am J Epidemiol. 2009;170:244–56.

12. Austin PC, Fine JP. Practical recommendations for reporting Fine-Gray model analyses for competing risk data. Stat Med. 2017;36:4391–400.
