# Supplementary material for: Competing risk events in antimalarial drug trials in uncomplicated Plasmodium falciparum malaria: a WorldWide Antimalarial Resistance Network individual participant data meta-analysis
Source: Malar J. 2019 Jul 5;18:225. doi: 10.1186/s12936-019-2837-4 (PMC6612160; doi:10.1186/s12936-019-2837-4)
Supplement: Supplementary file 3 — Additional file 3 Additional results. [file 12936_2019_2837_MOESM3_ESM.docx]

**Additional file 3:**

**Competing risk events in antimalarial drug trials in uncomplicated *Plasmodium falciparum* malaria: A WorldWide Antimalarial Resistance Network Individual Participant Data Meta-Analysis**

The WorldWide Antimalarial Resistance Network Methodology Study Group ^1^

^1^WorldWide Antimalarial Resistance Network (WWARN), Centre for Tropical Medicine and Global Health, Nuffield Department of Clinical Medicine, University of Oxford, Oxford, UK

Correspondences to

prabin.dahal@wwarn.org

[kasia.stepniewska@wwarn.org](mailto:kasia.stepniewska@wwarn.org)

1. **Additional information on the data included**

Table 1: Number of recrudescences, *P. falciparum* new infections and indeterminate recurrences stratified by sites and drug (only sites with > 25 observations shown)

| **WWARN ID** | **Day** | **Drug** | **Cured** | ***Pf* Recrudescence** | ***Pf* New Infection** | **Indeterminate outcomes** |
| --- | --- | --- | --- | --- | --- | --- |
| ACHGC.Kisumu | 28 | AL | 162 | 5 | 17 | 0 |
| AJXCU.Cameroon | 28 | AL | 50 | 0 | 0 | 0 |
| AJXCU.Cote D'Ivoire | 28 | AL | 77 | 1 | 0 | 2 |
| AJXCU.Senegal | 28 | AL | 76 | 0 | 0 | 0 |
| AKABP.Muheza | 28 | AL | 232 | 5 | 31 | 17 |
| ATMFH.Phuoc Long | 42 | DP | 45 | 9 | 1 | 0 |
| BQSYZ.KL | 28 | AL | 134 | 0 | 0 | 4 |
| CCEPC.Mbarara | 28 | AL | 920 | 1 | 27 | 5 |
| CDCMJ.Rakhine | 63 | ASAQ | 56 | 10 | 9 | 6 |
| CDCMJ.Rakhine | 63 | AL | 51 | 1 | 5 | 9 |
| CDCMJ.Rakhine | 63 | DP | 78 | 2 | 9 | 4 |
| CDCMJ.Rakhine | 63 | ASMQ | 86 | 0 | 4 | 3 |
| CDTEN.CS Baraka | 42 | AL | 122 | 3 | 14 | 4 |
| CDTEN.CS Baraka | 42 | ASAQ | 113 | 6 | 21 | 0 |
| CRAXS.Phuoc Chien | 42 | DP | 58 | 0 | 2 | 0 |
| CUDNY.Multi-site | 63 | DP | 164 | 5 | 0 | 13 |
| CXJYT.MOCK_CEN_07 | 28 | AL | 99 | 0 | 4 | 0 |
| CXJYT.MOCK_CEN_09 | 28 | AL | 84 | 7 | 16 | 0 |
| CXJYT.MOCK_CEN_15 | 28 | AL | 93 | 4 | 3 | 0 |
| DAABT.Gaya | 28 | AL | 67 | 2 | 9 | 1 |
| DAABT.Gaya | 28 | ASAQ | 77 | 2 | 1 | 0 |
| DADPZ.Tororo | 28 | AL | 91 | 0 | 22 | 0 |
| DADPZ.Tororo | 28 | DP | 115 | 0 | 4 | 0 |
| DAXCM.Mbita | 42 | AL | 123 | 4 | 23 | 3 |
| DAXCM.Mbita | 42 | DP | 140 | 0 | 3 | 2 |
| DBCXT.Tororo | 28 | AL | 142 | 0 | 1 | 1 |
| DFUZB.Burkina Faso | 42 | DP | 340 | 4 | 35 | 1 |
| DJYCQ.Bobo-Dioulasso | 28 | AL | 220 | 6 | 33 | 2 |
| DTDDU.DAKAR | 28 | ASAQ | 99 | 0 | 0 | 0 |
| DTDDU.KAOLACK | 28 | ASAQ | 63 | 2 | 0 | 6 |
| DTDDU.PODOR | 28 | ASAQ | 89 | 0 | 0 | 0 |
| DTDDU.RICHARD-TOLL | 28 | ASAQ | 42 | 0 | 0 | 0 |
| DTDDU.Senegal | 28 | ASMQ | 140 | 0 | 0 | 3 |
| DTDDU.VELINGARA | 28 | AL | 29 | 0 | 0 | 0 |
| DTDDU.VELINGARA | 28 | ASAQ | 52 | 0 | 0 | 0 |
| DYFKY.Nangarhar | 42 | DP | 41 | 0 | 0 | 0 |
| EDBXP.KICUKIRO | 28 | DP | 72 | 1 | 2 | 0 |
| EDBXP.MASHESHA | 28 | DP | 84 | 1 | 2 | 0 |
| EDBXP.RUKARA | 28 | DP | 73 | 9 | 8 | 0 |
| EDPJN.MOCK_CEN_01 | 42 | AL | 29 | 0 | 0 | 0 |
| EDPJN.MOCK_CEN_04 | 42 | AL | 97 | 0 | 23 | 1 |
| EDPJN.MOCK_CEN_06 | 42 | AL | 22 | 0 | 9 | 0 |
| EDPJN.MOCK_CEN_08 | 42 | AL | 97 | 0 | 10 | 3 |
| EDPJN.MOCK_CEN_11 | 42 | AL | 21 | 2 | 14 | 1 |
| EDPJN.MOCK_CEN_12 | 42 | AL | 209 | 8 | 13 | 10 |
| EDPJN.MOCK_CEN_14 | 42 | AL | 158 | 13 | 52 | 2 |
| EDPJN.MOCK_CEN_16 | 42 | AL | 73 | 5 | 24 | 0 |
| EFTTU.Multi-site | 28 | AL | 227 | 5 | 2 | 0 |
| EGYMA.Sikasso MALI | 28 | AL | 435 | 6 | 216 | 23 |
| EPDUY.MKT | 63 | DP | 62 | 0 | 6 | 0 |
| EPDUY.MLA | 63 | DP | 69 | 0 | 2 | 0 |
| EPDUY.MRC | 63 | DP | 29 | 0 | 2 | 0 |
| FAJXQ.1 | 28 | ASAQ | 94 | 3 | 5 | 3 |
| FAJXQ.2 | 28 | ASAQ | 95 | 2 | 0 | 0 |
| FEGFG.Assam | 28 | AL | 53 | 0 | 0 | 0 |
| FEGFG.Orissa | 28 | AL | 69 | 1 | 1 | 0 |
| FEKFF.Siaya | 42 | AL | 71 | 5 | 48 | 12 |
| FEKFF.Siaya | 42 | DP | 85 | 4 | 43 | 4 |
| FFNAU.Iquitos | 63 | DP | 240 | 2 | 4 | 6 |
| FSFKM.Mwanza | 28 | AL | 103 | 1 | 4 | 0 |
| GARJK.Uige | 28 | AL | 92 | 2 | 5 | 0 |
| GARJK.Uige | 28 | DP | 100 | 0 | 0 | 0 |
| GARJK.Zaire | 28 | AL | 84 | 7 | 9 | 0 |
| GARJK.Zaire | 28 | DP | 99 | 0 | 1 | 0 |
| GHNKU.MOCK_CEN_13 | 28 | AL | 153 | 4 | 1 | 2 |
| GPXJK.bfnanoro | 28 | AL | 119 | 24 | 142 | 9 |
| GPXJK.bfnanoro | 28 | ASAQ | 204 | 9 | 74 | 8 |
| GPXJK.bfnanoro | 28 | DP | 190 | 7 | 22 | 2 |
| GPXJK.gafougam | 28 | AL | 74 | 0 | 3 | 1 |
| GPXJK.gafougam | 28 | DP | 68 | 0 | 0 | 0 |
| GPXJK.gafougam | 28 | ASAQ | 72 | 2 | 4 | 1 |
| GPXJK.mzmanhic | 28 | DP | 198 | 3 | 5 | 2 |
| GPXJK.mzmanhic | 28 | ASAQ | 170 | 6 | 24 | 6 |
| GPXJK.ngikot | 28 | AL | 166 | 1 | 6 | 1 |
| GPXJK.ngikot | 28 | DP | 141 | 0 | 3 | 2 |
| GPXJK.ngikot | 28 | ASAQ | 163 | 0 | 8 | 3 |
| GPXJK.rwmashes | 28 | AL | 75 | 1 | 0 | 0 |
| GPXJK.rwmashes | 28 | DP | 71 | 0 | 4 | 1 |
| GPXJK.rwrukara | 28 | AL | 65 | 3 | 4 | 0 |
| GPXJK.rwrukara | 28 | DP | 69 | 2 | 0 | 0 |
| GPXJK.ugjinja | 28 | DP | 216 | 3 | 4 | 0 |
| GPXJK.ugjinja | 28 | AL | 213 | 4 | 5 | 3 |
| GPXJK.ugmbarar | 28 | DP | 150 | 1 | 5 | 3 |
| GPXJK.ugmbarar | 28 | ASAQ | 138 | 2 | 17 | 2 |
| GPXJK.ugtororo | 28 | AL | 124 | 8 | 75 | 14 |
| GPXJK.ugtororo | 28 | DP | 165 | 9 | 43 | 3 |
| GPXJK.zmndola | 28 | AL | 74 | 1 | 8 | 1 |
| GPXJK.zmndola | 28 | ASAQ | 84 | 0 | 1 | 0 |
| GPXJK.zmndola | 28 | DP | 76 | 0 | 0 | 0 |
| GTEFH.Khagrachari hill | 42 | AL | 101 | 0 | 0 | 20 |
| GZQDA.1 | 42 | AL | 143 | 8 | 100 | 7 |
| GZQDA.99 | 42 | AL | 75 | 1 | 23 | 2 |
| HCEMT.MOCK_CEN_03 | 42 | AL | 158 | 1 | 0 | 0 |
| HJNDX.Grand Gedeh County | 28 | ASAQ | 89 | 0 | 10 | 3 |
| HKNHR.Savannakhet | 42 | AL | 97 | 0 | 7 | 4 |
| HMPBZ.SUDAN | 28 | DP | 46 | 0 | 0 | 0 |
| JGGNM.31 | 42 | DP | 143 | 18 | 39 | 1 |
| JGGNM.31 | 42 | AL | 54 | 8 | 36 | 2 |
| JGGNM.33 | 42 | DP | 241 | 20 | 25 | 11 |
| JGGNM.33 | 42 | AL | 120 | 2 | 17 | 6 |
| JGGNM.34 | 42 | AL | 73 | 3 | 13 | 2 |
| JGGNM.34 | 42 | DP | 169 | 2 | 11 | 1 |
| JGGNM.35 | 42 | AL | 68 | 4 | 26 | 2 |
| JGGNM.35 | 42 | DP | 163 | 9 | 30 | 1 |
| JTXEY.Kampala | 28 | AL | 83 | 0 | 3 | 0 |
| JXZNZ.Bobo Dioulasso | 28 | AL | 319 | 8 | 69 | 3 |
| JXZNZ.Bobo Dioulasso | 28 | ASAQ | 345 | 1 | 41 | 0 |
| JXZNZ.Gourcy | 28 | AL | 101 | 12 | 28 | 3 |
| JXZNZ.Gourcy | 28 | ASAQ | 119 | 8 | 14 | 2 |
| KJGJT.Koupela | 28 | ASAQ | 411 | 8 | 18 | 2 |
| KPSGN.Chittagong | 42 | AL | 59 | 3 | 5 | 0 |
| KRBXE.Dabhine | 42 | DP | 183 | 2 | 2 | 1 |
| KRBXE.Mingan | 42 | DP | 131 | 0 | 0 | 0 |
| KZBZT.Mbita | 28 | DP | 73 | 0 | 0 | 0 |
| KZBZT.Mbita | 28 | AL | 72 | 0 | 1 | 0 |
| MCZHT.Kanungu | 42 | AL | 135 | 9 | 49 | 6 |
| MCZHT.Kanungu | 42 | DP | 189 | 4 | 21 | 1 |
| MEFSC.Ghana: Kintampo | 42 | AL | 22 | 1 | 38 | 2 |
| MEFSC.Kenya: Eldoret | 42 | AL | 42 | 2 | 7 | 1 |
| MEFSC.Nigeria: Calabar | 42 | AL | 36 | 2 | 4 | 6 |
| MEFSC.Nigeria: Enugu | 42 | AL | 49 | 1 | 22 | 2 |
| MEFSC.Nigeria: Jos | 42 | AL | 37 | 0 | 0 | 1 |
| MEFSC.Tanzania: Kiwangwa | 42 | AL | 85 | 0 | 10 | 1 |
| MPYTS.Xepon | 42 | AL | 487 | 20 | 25 | 17 |
| MTZZP.Phalanxay | 42 | DP | 104 | 0 | 3 | 0 |
| NBSAE.Kambila | 28 | AL | 159 | 7 | 66 | 3 |
| NJUCK.1 | 28 | AL | 44 | 0 | 0 | 2 |
| NJUCK.2 | 28 | ASAQ | 41 | 0 | 0 | 1 |
| NJUCK.6 | 28 | AL | 27 | 0 | 1 | 1 |
| NJUCK.6 | 28 | ASAQ | 28 | 1 | 0 | 1 |
| PCPRC.Gedaref | 28 | AL | 50 | 1 | 0 | 2 |
| PCPRC.New Halfa | 28 | AL | 31 | 0 | 2 | 0 |
| PFJCR.1 | 28 | ASAQ | 26 | 0 | 0 | 0 |
| PKGFP.Congo-Kindamba | 28 | AL | 92 | 0 | 4 | 10 |
| PUEKP.Kisumu | 28 | AL | 237 | 14 | 16 | 0 |
| QRBRC.Pweto | 42 | ASAQ | 141 | 3 | 10 | 1 |
| QRBRC.Pweto | 42 | AL | 123 | 1 | 19 | 1 |
| QXJGK.11 | 63 | DP | 46 | 0 | 1 | 0 |
| QXJGK.12 | 63 | DP | 91 | 1 | 9 | 1 |
| QXJGK.19 | 63 | DP | 84 | 1 | 3 | 0 |
| QXJGK.20 | 63 | DP | 143 | 1 | 9 | 0 |
| QXJGK.21 | 63 | DP | 67 | 3 | 29 | 1 |
| QXJGK.22 | 63 | DP | 129 | 5 | 30 | 2 |
| QXJGK.41 | 63 | DP | 44 | 0 | 0 | 0 |
| QXJGK.43 | 63 | DP | 33 | 1 | 1 | 1 |
| QZJGM.Pingilikani | 63 | AL | 139 | 23 | 52 | 3 |
| QZJGM.Pingilikani | 63 | DP | 145 | 15 | 47 | 3 |
| QZMAG.Hanura | 42 | DP | 179 | 0 | 1 | 0 |
| RDBXS.BANDIAGARA | 28 | AL | 72 | 3 | 23 | 1 |
| RDBXS.FALADJE | 28 | AL | 60 | 3 | 24 | 0 |
| RDBXS.KOLLE | 28 | AL | 51 | 1 | 22 | 3 |
| RDBXS.PONGONON | 28 | AL | 66 | 1 | 4 | 0 |
| REAJS.Keur Sosse | 28 | ASAQ | 175 | 0 | 1 | 4 |
| REAJS.Keur Sosse | 28 | AL | 165 | 0 | 4 | 5 |
| RMRNH.BurkinaFaso | 63 | AL | 64 | 5 | 58 | 1 |
| RMRNH.BurkinaFaso | 63 | ASMQ | 65 | 2 | 59 | 1 |
| RMRNH.BurkinaFaso4 | 63 | ASMQ | 30 | 5 | 31 | 0 |
| RMRNH.BurkinaFaso4 | 63 | AL | 23 | 3 | 37 | 1 |
| RMRNH.Kenya | 63 | ASMQ | 63 | 4 | 23 | 9 |
| RMRNH.Kenya | 63 | AL | 55 | 5 | 27 | 6 |
| RMRNH.Kenya2 | 63 | AL | 42 | 1 | 18 | 11 |
| RMRNH.Kenya2 | 63 | ASMQ | 32 | 3 | 28 | 10 |
| RMRNH.Tanzania2 | 63 | AL | 21 | 1 | 3 | 2 |
| RMRNH.Tanzania2 | 63 | ASMQ | 20 | 0 | 7 | 1 |
| RMRNH.Tanzania3 | 63 | AL | 40 | 1 | 13 | 2 |
| RMRNH.Tanzania3 | 63 | ASMQ | 38 | 0 | 13 | 4 |
| RNZFN.CI | 28 | AL | 79 | 0 | 0 | 0 |
| RNZFN.SEN | 28 | AL | 77 | 0 | 0 | 5 |
| RSBPS.Anonkoua-koute | 28 | AL | 59 | 0 | 0 | 1 |
| SATNJ.Ayame | 28 | ASAQ | 59 | 0 | 0 | 2 |
| SATNJ.Ayame | 28 | AL | 59 | 0 | 0 | 1 |
| SATNJ.Dabakala | 28 | ASAQ | 62 | 0 | 0 | 0 |
| SATNJ.Dabakala | 28 | AL | 56 | 0 | 0 | 3 |
| SBCEE.1 | 42 | AL | 67 | 4 | 128 | 6 |
| SBCEE.1 | 42 | ASAQ | 70 | 2 | 130 | 5 |
| SFBSG.MKT | 63 | AL | 81 | 2 | 25 | 1 |
| SFBSG.MLA | 42 | AL | 83 | 5 | 0 | 0 |
| SUEGP.Bobo-Dioulasso | 42 | DP | 176 | 2 | 9 | 0 |
| SUEGP.Bobo-Dioulasso | 42 | AL | 135 | 5 | 48 | 0 |
| SXGQP.Bandim | 42 | AL | 67 | 3 | 1 | 0 |
| SXGQP.Belem | 42 | AL | 51 | 0 | 1 | 0 |
| SXGQP.Cuntum | 42 | AL | 62 | 1 | 0 | 1 |
| SYFQT.Bagamoyo | 42 | AL | 12 | 9 | 29 | 0 |
| SZRDK.MKT | 63 | ASMQ | 38 | 2 | 12 | 2 |
| SZRDK.MLA | 63 | ASMQ | 38 | 0 | 1 | 2 |
| SZRDK.MRC | 63 | ASMQ | 28 | 0 | 2 | 0 |
| SZRDK.WPA | 63 | ASMQ | 48 | 9 | 10 | 4 |
| TDFKY.Apac | 42 | DP | 119 | 0 | 77 | 15 |
| TDFKY.Apac | 42 | AL | 91 | 0 | 79 | 40 |
| TRKFC.Agades | 28 | AL | 38 | 3 | 1 | 0 |
| TRKFC.Gaya | 28 | AL | 71 | 7 | 2 | 0 |
| TRKFC.Tessaoua | 28 | AL | 54 | 5 | 11 | 0 |
| TYKSC.11 | 28 | AL | 57 | 1 | 2 | 0 |
| TYKSC.11 | 28 | ASAQ | 117 | 0 | 2 | 0 |
| TYKSC.21 | 28 | AL | 56 | 0 | 0 | 0 |
| TYKSC.21 | 28 | ASAQ | 109 | 0 | 1 | 0 |
| TYKSC.31 | 28 | AL | 41 | 2 | 22 | 3 |
| TYKSC.31 | 28 | ASAQ | 95 | 2 | 37 | 1 |
| TYKSC.41 | 28 | ASAQ | 174 | 0 | 11 | 2 |
| TYKSC.41 | 28 | AL | 78 | 1 | 10 | 0 |
| TYKSC.51 | 28 | AL | 38 | 1 | 0 | 0 |
| TYKSC.51 | 28 | ASAQ | 77 | 0 | 0 | 0 |
| UANQM.Madang | 42 | DP | 57 | 4 | 18 | 1 |
| UANQM.Madang | 42 | AL | 32 | 2 | 20 | 0 |
| UBTXH.LIBERIA | 42 | AL | 87 | 7 | 45 | 6 |
| UBTXH.LIBERIA | 42 | ASAQ | 78 | 3 | 65 | 2 |
| UGPAG.Mbita | 28 | AL | 72 | 1 | 1 | 1 |
| XEDNN.1 | 28 | AL | 45 | 1 | 2 | 0 |
| XEDNN.1 | 28 | DP | 54 | 1 | 1 | 0 |
| XEDNN.2 | 28 | DP | 75 | 0 | 0 | 0 |
| XEDNN.2 | 28 | AL | 69 | 1 | 1 | 0 |
| XEDNN.3 | 28 | DP | 66 | 0 | 0 | 0 |
| XEDNN.3 | 28 | AL | 68 | 0 | 0 | 0 |
| XEKED.Kaolack | 28 | ASMQ | 158 | 0 | 0 | 0 |
| XEKED.Kaolack | 28 | AL | 154 | 0 | 0 | 6 |
| XTGNB.Bulbula | 42 | AL | 99 | 0 | 0 | 1 |
| XXFCZ.Kibaha | 42 | AL | 107 | 15 | 114 | 8 |
| YGNQS.Pikine GuÃ©diawaye | 42 | DP | 123 | 1 | 0 | 0 |
| YGNQS.Pikine GuÃ©diawaye | 42 | AL | 128 | 3 | 8 | 0 |
| YGNQS.Pikine GuÃ©diawaye | 42 | ASAQ | 124 | 3 | 3 | 0 |
| YGTAH.Tororo | 28 | AL | 103 | 10 | 89 | 2 |
| YPRHD.MKT | 42 | AL | 144 | 9 | 30 | 20 |
| YPRHD.MLA | 42 | AL | 194 | 5 | 16 | 1 |
| YRRSK.Angola-Caala | 28 | AL | 66 | 0 | 1 | 1 |
| YYDSM.A | 42 | AL | 36 | 4 | 4 | 1 |
| YYDSM.A | 42 | ASAQ | 33 | 3 | 10 | 0 |
| YYDSM.S | 42 | AL | 37 | 3 | 6 | 1 |
| YYDSM.S | 42 | ASAQ | 27 | 8 | 12 | 0 |
| ZHKTN.LIBERIA | 28 | AL | 110 | 1 | 0 | 0 |
| ZMNBX.Dac O | 63 | DP | 1727 | 19 | 87 | 11 |
| ZMNBX.Dac O | 42 | ASMQ | 94 | 4 | 0 | 1 |
| ZYBXE.Kivunge | 42 | AL | 110 | 10 | 27 | 2 |
| ZYBXE.Micheweni | 42 | AL | 46 | 0 | 4 | 1 |

AL= Artmether-Lumefantrine; ASAQ= Artesunate-amodiaquine; ASMQ= Artesunate-mefloquine; DP= Dihydroartemisinin-piperaquine

## **Comparative efficacy studies**

Comparing Artemether-Lumefantrine (AL) against Dihdroartesiminin-Piperaquine (DP) using log-rank test and Gray’s *k*-sample test

| **Study (site)** | **Day** | ${\hat{\boldsymbol{F}}}_{\boldsymbol{CIF}}$ **estimate of recrudescence**  **[95% confidence interval]** | | ${\hat{\boldsymbol{F}}}_{\boldsymbol{KM}}$ **estimate of recrudescence**  **[95% confidence interval]** | | **Tests for equality of failure**  **(*P*-value)** | |
| --- | --- | --- | --- | --- | --- | --- | --- |
|  |  | **AL** | **DP** | **AL** | **DP** | **Log-rank**  **test** | **Gray's *k*-sample**  **test** |
| Arinaitwe-2009 (Uganda, Tororo) [1] | 28 | 0.0% | 0.0% | 0.0% | 0.0% | - | - |
| Sawa-2013 (Kenya, Mbita) [2] | 42 | 2.7% [0.1 - 5.4] | 0.0% | 2.8% [0.1 - 5.5] | 0.0% | 0.050 | 0.053 |
| Agrawal-2013 (Kenya,Siaya) [3] | 42 | 4.2% [0.6 - 7.8] | 3.6% [0.1 – 7.0] | 5.2% [0.5 – 10.0] | 4.7% [0.2 - 9.3] | 0.550 | 0.707 |
| The 4ABC Trial (Burkina Faso, Nanoro) [4] | 28 | 8.6% [5.3 - 11.8] | 3.2% [0.9 - 5.6] | 11.1% [6.8 - 15.4] | 3.3% [0.9 - 5.7] | 0.001 | 0.012 |
| The 4ABC Trial (Gabon, Fougamou) [4] | 28 | 0.0% | 0.0% | 0.0% | 0.0% | - | - |
| The 4ABC Trial (Nigeria,Afokang) [4] | 28 | 0.7% [0.0 - 1.9] | 0.0% | 0.7% [0.0 - 2.0] | 0.0% | 0.372 | 0.666 |
| The 4ABC Trial (Rwanda, Mashesha) [4] | 28 | 1.3% [0.0 - 3.9] | 0.0% | 1.3% [0.0 - 3.9] | 0.0% | 0.321 | 0.317 |
| The 4ABC Trial (Rwanda, Rukara) [4] | 28 | 4.2% [0.0 - 8.9] | 2.8% [0.0 - 6.7] | 4.2% [0.0 - 8.9] | 2.8% [0.0- 6.7] | 0.643 | 0.648 |
| The 4ABC Trial (Uganda, Jinja) [4] | 28 | 1.9% [0.1 - 3.8] | 1.4% [0.0 - 3] | 2.0% [0.1 - 3.9] | 1.4% [0.0 – 3.0] | 0.716 | 0.727 |
| The 4ABC Trial (Uganda, Tororo) [4] | 28 | 3.7% [1.2 - 6.2] | 4.2% [1.5 - 6.8] | 4.1% [1.3 - 7.0] | 4.3% [1.6 - 7.1] | 0.996 | 0.819 |
| The 4ABC Trial (Zambia, Ndola) [4] | 28 | 1.4% [0.0 - 4.1] | 0.0% | 1.4% [0.0 - 4.1] | 0.0% | 0.331 | 0.331 |
| Adam 2010 (Sudan,Sinnar) | 28 | 0.0% | 0.0% | 0.0% | 0.0% | - | - |
| Bassat-2009 (Burkina Faso, Nanoro) [5] | 42 | 8.1% [2.7 - 13.5] | 9.1% [5.1 - 13.1] | 9.9% [3.2 - 16.6] | 9.8% [5.5 - 14.1] | 0.675 | 0.828 |
| Bassat-2009 (Mozambique, Manhica) [5] | 42 | 1.5% [0.0 - 3.6] | 6.9% [4.0 - 9.9] | 1.6% [0.0 - 3.8] | 7.2% [4.1 - 10.2] | 0.021 | 0.018 |
| Bassat-2009 (Uganda,Mbarara) [5] | 42 | 3.4% [0.0 - 7.2] | 1.1% [0.0- 2.6] | 3.6% [0.0 - 7.7] | 1.1% [0.0 - 2.6] | 0.189 | 0.204 |
| Bassat-2009 (Zambia, Ndola) [5] | 42 | 4.1% [0.1 - 8.1] | 4.6% [1.7 - 7.6] | 4.4% [0.2 - 8.7] | 4.9% [1.8 - 7.9] | 0.931 | 0.909 |
| Mens-2008 (Kenya, Mbita) [6] | 28 | 0.0% | 0.0% | 0.0% | 0.0% | - | - |
| Yeka-2008 (Uganda,Kanungu) [7] | 42 | 4.6% [1.7 - 7.6] | 1.9% [0.1 - 3.7] | 5.2% [1.9 - 8.5] | 1.9% [0.0 - 3.8] | 0.082 | 0.118 |
| Borrmann-2011 (Kenya, Pingilikani) [8] | 63 | 11.4% [7.0 - 15.7] | 7.8% [4.0 - 11.7] | 12.9% [8.0 - 17.9] | 8.7% [4.4 - 12.9] | 0.237 | 0.251 |
| Zongo-2007b (Burkina Faso, Bobo) [9] | 42 | 2.8% [0.4 - 5.3] | 1.2% [0.0 - 2.8] | 3.1% [0.4 - 5.8] | 1.2% [0.0 - 2.8] | 0.246 | 0.274 |
| Kamya-2007 (Uganda, Apac) [10] | 42 | 0.0% | 0.0% | 0.0% | 0.0% | - | - |
| Karunajeewa-2008 (PNG, Madang) [11] | 42 | 4.7% [0.0 - 11.3] | 6.5% [0.2 - 12.7] | 5.9% [0.0 - 14.2] | 7.1% [0.3 - 13.9] | 0.825 | 0.753 |
| Karunajeewa-2008 (PNG, Maprik) [11] | 42 | 14.3% [0.0 - 33.3] | 18.2% [1.7 - 34.7] | 14.3% [0.0 - 32.6] | 18.2% [2.1 - 34.3] | 0.763 | 0.763 |
| Yavo-2011 (Cameroon,Yaounde) [12] | 28 | 2.1% [0.0 - 6.2] | 1.8% [0.0 - 5.3] | 2.1% [0.0 - 6.1] | 1.8% [0.0 - 5.3] | 0.913 | 0.913 |
| Yavo-2011 (Ivorycoast, Bocabo) [12] | 28 | 1.4% [0.0 - 4.2] | 0.0% | 1.4% [0.0 - 4.2] | 0.0% | 0.307 | 0.307 |
| Yavo-2011 (Senegal,Touba) [12] | 28 | 0.0% | 0.0% | 0.0% | 0.0% | - | - |
| Sylla-2013 (Senegal, Pikine) [13] | 42 | 2.3% [0.0 - 4.8] | 0.9% [0.0 - 2.6] | 2.3% [0.0 - 4.9] | 0.9% [0.0 - 2.6] | 0.372 | 0.384 |

$\hat{F}_{KM}\left( t \right)$= Cumulative failure estimates derived using 1- Kaplan-Meier approach; $\hat{F}_{CIF}\left( t \right)$= Cumulative failure estimates derived using Cumulative Incidence Function; AL= artemether-lumefantrine; DP= dihydroartemisinin-piperaquine; PNG = Papua New Guinea

**Comparing artemether-lumefantrine against artesunate-mefloquine using log-rank test and Gray’s *k*-sample test**

|  |  | ${\hat{\boldsymbol{F}}}_{\boldsymbol{CIF}}$ **estimate of recrudescence**  **(95% confidence interval)** | | ${\hat{\boldsymbol{F}}}_{\boldsymbol{KM}}$ **estimate of recrudescence**  **(95% confidence interval)** | | **Tests for equality of failure**  **(*P*-value)** | |
| --- | --- | --- | --- | --- | --- | --- | --- |
| **Study (site)** | **Day** | **AL** | **ASMQ** | **AL** | **ASMQ** | **Log-rank**  **test** | **Gray's *k*-sample**  **test** |
| Smithuis-2010 (Myanmar, Rakhine) [14] | 63 | 1.3% [0.0 - 3.9] | 0.0% | 1.4% [0.0 - 4.0] | 0.0% | 0.220 | 0.221 |
| Faye-2007 (Senegal, multisite) [15] | 28 | 0.0% | 0.0% | 0.0% | 0.0% | - | - |
| Sirima-2015 (Burkina Faso, Balonghin) [16] | 63 | 4.0% [0.6 - 7.5] | 1.6% [0.0 - 3.9] | 5.4% [0.7 - 10.2] | 2.1% [0.0 - 5.0] | 0.188 | 0.255 |
| Sirima-2015 (Burkina Faso, Banfora) [16] | 63 | 5.1% [0.0 - 10.8] | 8.1% [1.2 - 14.9] | 8.5% [0.0 - 18.1] | 11.5% [1.7 - 21.4] | 0.699 | 0.515 |
| Sirima-2015 (Kenya, Kisumu) [16] | 63 | 6.4% [0.9 - 11.9] | 4.8% [0.2 - 9.4] | 8.6% [1.2 - 16.1] | 6.1% [0.1 - 12] | 0.507 | 0.614 |
| Sirima-2015 (Kenya, Ahero) [16] | 63 | 1.8% [0.0 - 5.3] | 4.7% [0.0 - 10.0] | 2.9% [0.0 - 8.6] | 7.8% [0.0 - 16.7] | 0.375 | 0.385 |
| Sirima-2015 (Tanzania, Kilosa) [16] | 63 | 0.0% | 5.3% [0.0 - 15.6] | 0.0% | 5.6% [0.0 - 16.2] | 0.361 | 0.331 |
| Sirima-2015 (Tanzania, Korogwe) [16] | 63 | 4.3% [0.0 - 12.9] | 0.0% | 5.0% [0.0 - 14.5] | 0.0% | 0.371 | 0.343 |
| Sirima-2015 (Tanzania, Bagamayo) [16] | 63 | 2% [0.0 - 5.9] | 0.0% | 2.8% [0.0 - 8.2] | 0.0% | 0.405 | 0.985 |
| Faye-2010 (Senegal, Kaolack) [17] | 28 | 0.0% | 0.0% | 0.0% | 0.0% | - | - |

$\hat{F}_{KM}\left( t \right)$= Cumulative failure estimates derived using 1- Kaplan-Meier approach

$\hat{F}_{CIF}\left( t \right)$= Cumulative failure estimates derived using Cumulative Incidence Function

AL= artemether-lumefantrine; ASMQ= artesunate-mefloquine

Comparing artemether-lumefantrine against artesunate-amodiquine using log-rank test and Gray’s k-sample test

| **Study (site)** | **Day** | ${\hat{\boldsymbol{F}}}_{\boldsymbol{CIF}}$ **estimate of recrudescence**  **[95% confidence interval]** | | ${\hat{\boldsymbol{F}}}_{\boldsymbol{KM}}$ **estimate of recrudescence**  **[95% confidence interval]** | | **Tests for equality of failure**  **(*P*-value)** | |
| --- | --- | --- | --- | --- | --- | --- | --- |
|  |  | **AL** | **ASAQ** | **AL** | **ASAQ** | **Log-rank**  **test** | **Gray's *k*-sample**  **test** |
| Smithuis-2010 (Myanmar, Rakhine) [14] | 63 | 1.3% [0.0 - 3.9] | 11.8% [5.2 - 18.4] | 1.4% [0.0- 4.0] | 12.3% [5.5 - 19.1] | 0.008 | 0.008 |
| de Wit-2015* (DRC, Baraka) | 42 | 2.4% [0.0 - 5.1] | 5% [1.1 - 9.0] | 2.6% [0.0 - 5.5] | 5.4% [1.2 - 9.7] | 0.270 | 0.281 |
| The 4ABC Trial (Burkina Faso, Nanoro) [4] | 28 | 8.6% [5.3 - 11.8] | 3.1% [1.1 - 5.2] | 11.1% [6.8 - 15.4] | 3.5% [1.2 - 5.7] | 0.001 | 0.006 |
| The 4ABC Trial (Gabon, Fougamou) [4] | 28 | 0.0% | 2.8% [0.0 - 6.7] | 0.0% | 2.9% [0 - 6.8] | 0.154 | 0.159 |
| The 4ABC Trial (Nigeria,Afokang) [4] | 28 | 0.7% [0.0 - 1.9] | 0.0% | 0.7% [0.0 - 2.0] | 0.0% | 0.320 | 0.313 |
| The 4ABC Trial (Zambia, Ndola) [4] | 28 | 1.4% [0.0 - 4.1] | 0.0% | 1.4% [0.0 - 4.1] | 0.0% | 0.328 | 0.328 |
| Nikiema-2010* (Burkina Faso, Bobo) | 28 | 2.1% [0.7 - 3.5] | 0.3% [0.0 - 0.8] | 2.2% [0.7 - 3.7] | 0.3% [0.0 - 0.9] | 0.019 | 0.021 |
| Nikiema-2010* (Burkina Faso, Gourcy) | 28 | 9.4% [4.3 - 14.5] | 5.7% [1.9 - 9.6] | 10.3% [4.7 - 15.8] | 5.8% [1.9 - 9.7] | 0.225 | 0.281 |
| Espié-2012 (DRC, Pweto) [18] | 42 | 0.8% [0.0 - 2.3] | 2.2% [0.0 - 4.7] | 0.8% [0.0 - 2.4] | 2.3% [0.0 - 4.8] | 0.366 | 0.336 |
| Ndiaye-2011 (Senegal, Keur Sosse) [19] | 28 | 0.0% | 0.0% | 0.0% | 0.0% | - | - |
| Offianan-2011 (Ivorycoast, Ayame) [20] | 28 | 0.0% | 0.0% | 0.0% | 0.0% | - | - |
| Offianan-2011 (Ivorycoast, Dabakala) [20] | 28 | 0.0% | 0.0% | 0.0% | 0.0% | - | - |
| Yeka-2014 (Uganda, Tororo) [21] | 42 | 2.0% [0.1 - 3.9] | 1.0% [0.0 - 2.4] | 2.3% [0.1 - 4.6] | 1.0% [0.0 - 2.5] | 0.420 | 0.423 |
| Ndiaye-2009 (Madagascar,Tsiroanomandidy) [22] | 28 | 1.7% [0.0 - 4.9] | 0.0% | 1.7% [0.0 - 4.9] | 0.0% | 0.166 | 0.166 |
| Ndiaye-2009 (Cameroon,Yaoundé) [22] | 28 | 0.0% | 0.0% | 0.0% | 0.0% | - | - |
| Ndiaye-2009 (Mali, Bancoumana) [22] | 28 | 3.1% [0.0 - 7.5] | 1.6% [0.0- 3.7] | 3.7% [0.0 - 8.8] | 1.7% [0.0 - 4.1] | 0.427 | 0.472 |
| Ndiaye-2009 (Senegal, Mlomp/Keur-Socé) [22] | 28 | 1.3% [0.0 - 3.9] | 0.0% | 1.5% [0.0 - 4.3] | 0.0% | 0.150 | 0.161 |
| Ndiaye-2009 (Senegal, Mlomp/Keur-Socé) [22] | 28 | 2.6% [0.0 - 7.8] | 0.0% | 2.6% [0.0 - 7.7] | 0.0% | 0.175 | 0.175 |
| Schramm-2013 (Liberia, Nimba) [23] | 42 | 5% [1.4 - 8.6] | 2.1% [0.0 - 4.4] | 5.6% [1.6 - 9.7] | 2.7% [0.0 - 5.9] | 0.255 | 0.177 |
| Sylla-2013 (Senegal, Pikine) [13] | 42 | 2.3% [0.0- 4.8] | 2.5% [0.0 - 5.3] | 2.3% [0.0 - 4.9] | 2.5% [0.0 - 5.3] | 0.930 | 0.913 |
| Faucher-2009 (Benin, Allada) [24] | 42 | 10.1% [0.6 - 19.7] | 7.0% [0.0 - 14.7] | 10.4% [0.7 - 20.1] | 8.5% [0.0 - 17.7] | 0.624 | 0.555 |
| Faucher-2009 (Benin, Sekou) [24] | 42 | 7.0% [0.0- 14.7] | 18.2% [6.7 - 29.7] | 7.2% [0.0 - 15.1] | 20.7% [7.8 - 33.6] | 0.077 | 0.113 |

$\hat{F}_{KM}\left( t \right)$= Cumulative failure estimates derived using 1- Kaplan-Meier approach

$\hat{F}_{CIF}\left( t \right)$= Cumulative failure estimates derived using Cumulative Incidence Function; AL= artemether-lumefantrine; ASAQ= artesunate-amodiaquine

Comparing artesunate-amodiaquine against dihydroartemisinin-piperaquine using log-rank test and Gray’s k-sample test

| **Study (site)** | **Day** | ${\hat{\boldsymbol{F}}}_{\boldsymbol{CIF}}$ **estimate of recrudescence**  **[95% confidence interval]** | | ${\hat{\boldsymbol{F}}}_{\boldsymbol{KM}}$ **estimate of recrudescence**  **[95% confidence interval]** | | | **Tests for equality of failure**  **(*P*-value)** | | |
| --- | --- | --- | --- | --- | --- | --- | --- | --- | --- |
|  |  | **ASAQ** | **DP** | **ASAQ** | **DP** | **Log-rank**  **test** | | **Gray's**  ***k*-sample test** |  |
| Smithuis-2010 (Myanmar, Rakhine) [14] | 63 | 11.8% [5.2 - 18.4] | 1.9% [0.0 - 4.6] | 12.3% [5.5 - 19.1] | 2.0% [0.0 - 4.7] | 0.005 | | 0.005 |  |
| The 4ABC Trial (Burkina Faso, Nanoro) [4] | 28 | 3.1% [1.1 - 5.2] | 3.2% [0.9 - 5.6] | 3.5% [1.2 - 5.7] | 3.3% [0.9 - 5.7] | 0.824 | | 0.995 |  |
| The 4ABC Trial (Gabon, Fougamou) [4] | 28 | 2.8% [0.0 - 6.7] | 0.0% | 2.9% [0.0 - 6.8] | 0.0% | 0.180 | | 0.186 |  |
| The 4ABC Trial (Mozambique, Manhica) [4] | 28 | 3.2% [0.7 - 5.7] | 1.6% [0.0 - 3.4] | 3.3% [0.7 - 5.9] | 1.6% [0.0 - 3.4] | 0.284 | | 0.308 |  |
| The 4ABC Trial (Nigeria,Afokang) [4] | 28 | 0.0% | 0.0% | 0.0% | 0.0% | - | | - |  |
| The 4ABC Trial (Uganda,Mbarara) [4] | 28 | 1.3% [0.0 - 3.2] | 0.7% [0.0 - 2.0] | 1.4% [0.0 - 3.3] | 0.7% [0.0 - 2.0] | 0.554 | | 0.562 |  |
| The 4ABC Trial (Zambia, Ndola) [4] | 28 | 0.0% | 0.0% | 0.0% | 0.0% | - | | - |  |
| Sylla-2013 (Senegal, Pikine) [13] | 42 | 2.5% [0.0 - 5.3] | 0.9% [0.0 - 2.6] | 2.5% [0.0 - 5.3] | 0.9% [0.0 - 2.6] | 0.334 | | 0.339 |  |

$\hat{F}_{KM}\left( t \right)$= Cumulative failure estimates derived using 1- Kaplan-Meier approach

$\hat{F}_{CIF}\left( t \right)$= Cumulative failure estimates derived using Cumulative Incidence Function

ASAQ= artesunate-amodiaquine; DP= dihydroartemisinin-piperaquine

Comparing artesunate-mefloquine against dihydroartemisinin-piperaquine using log-rank test and Gray’s *k*-sample test

| **Study (site)** | **Day** | ${\hat{\boldsymbol{F}}}_{\boldsymbol{CIF}}$ **estimate of recrudescence**  **[95% confidence interval]** | | | ${\hat{\boldsymbol{F}}}_{\boldsymbol{KM}}$ **estimate of recrudescence**  **[95% confidence interval]** | | | **Tests for equality of failure**  **(*P*-value)** | | |
| --- | --- | --- | --- | --- | --- | --- | --- | --- | --- | --- |
|  |  | **ASMQ** | **DP** | **ASMQ** | | **DP** | **Log-rank**  **test** | | **Gray's *k*-sample**  **test** |  |
| Smithuis-2010 (Myanmar, Rakhine) [14] | 63 | 0% | 1.9% [0.0- 4.6] | 0% | | 2%[0.0 - 4.7] | 0.134 | | 0.137 |  |
| Hien-2006* (VietNam, Dac O) | 56 | 12.2% [0.0 - 24.5] | 2.7% [1.3 - 4.1] | 12.5% [0.2 - 24.8] | | 3% [1.5 - 4.5] | 0.001 | | 0.001 |  |

$\hat{F}_{KM}\left( t \right)$= Cumulative failure estimates derived using 1- Kaplan-Meier approach

$\hat{F}_{CIF}\left( t \right)$= Cumulative failure estimates derived using Cumulative Incidence Function

ASMQ= artesunate-mefloquine; DP= dihydroartemisinin-piperaquine

Comparing artesunate-mefloquine against artesunate-amodiaquine using log-rank test and Gray’s *k*-sample test

| **Study (site)** | **Day** | ${\hat{\boldsymbol{F}}}_{\boldsymbol{CIF}}$ **estimate of recrudescence**  **[95% confidence interval]** | | ${\hat{\boldsymbol{F}}}_{\boldsymbol{KM}}$ **estimate of recrudescence**  **[95% confidence interval]** | | **Tests for equality of failure**  **(*P*-value)** | |
| --- | --- | --- | --- | --- | --- | --- | --- |
|  |  | **ASMQ** | **ASAQ** | **ASMQ** | **ASAQ** | **Log-rank**  **test** | **Gray's *k*-sample**  **test** |
| Smithuis-2010 (Myanmar, Rakhine) [14] | 63 | 0.0% | 11.8% [5.2 - 18.4] | 0.0% | 12.3% [5.5 - 19.1] | *p*<0.001 | *p*<0.001 |

$\hat{F}_{KM}\left( t \right)$= Drug failure estimates derived using 1- Kaplan-Meier approach

$\hat{F}_{CIF}\left( t \right)$= Drug failure estimates derived using Cumulative Incidence Function

ASMQ= artesunate-mefloquine; ASAQ= artesunate-amodiaquine

**References**

1. Arinaitwe E, Sandison TG, Wanzira H, Kakuru A, Homsy J, Kalamya J, et al. Artemether-Lumefantrine versus Dihydroartemisinin- Piperaquine for Falciparum Malaria: A Longitudinal, Randomized Trial in Young Ugandan Children. Clin Infect Dis. 2009;49:1629–37.

2. Sawa P, Shekalaghe SA, Drakeley CJ, Sutherland CJ, Mweresa CK, Baidjoe AY, et al. Malaria transmission after artemether-lumefantrine and dihydroartemisinin- piperaquine: A randomized trial. J Infect Dis. 2013;207:1637–45.

3. Agarwal A, McMorrow M, Onyango P, Otieno K, Odero C, Williamson J, et al. A randomized trial of artemether-lumefantrine and dihydroartemisinin-piperaquine in the treatment of uncomplicated malaria among children in western Kenya. Malar J. 2013;12:254.

4. The Four Artemisinin-Based Combinations (4ABC) Study group. A head-to-head comparison of four artemisinin-based combinations for treating uncomplicated malaria in african children: A randomized trial. PLoS Med. 2011;8.

5. Bassat Q, Mulenga M, Tinto H, Piola P, Borrmann S, Menéndez C, et al. Dihydroartemisinin-piperaquine and artemether-lumefantrine for treating uncomplicated malaria in African children: A randomised, non-inferiority trial. PLoS One. 2009;4.

6. Mens PF, Sawa P, Van Amsterdam SM, Versteeg I, Omar SA, Schallig HDFH, et al. A randomized trial to monitor the efficacy and effectiveness by QT-NASBA of artemether-lumefantrine versus dihydroartemisinin-piperaquine for treatment and transmission control of uncomplicated Plasmodium falciparum malaria in western Kenya. Malar J. 2008;7:237.

7. Yeka A, Dorsey G, Kamya MR, Talisuna A, Lugemwa M, Rwakimari JB, et al. Artemether-lumefantrine versus dihydroartemisinin-piperaquine for treating uncomplicated malaria: a randomized trial to guide policy in Uganda. PLoS One. 2008;3:e2390.

8. Borrmann S, Sasi P, Mwai L, Bashraheil M, Abdallah A, Muriithi S, et al. Declining responsiveness of plasmodium falciparum infections to Artemisinin-Based combination treatments on the Kenyan coast. PLoS One. 2011;6.

9. Zongo I, Dorsey G, Rouamba N, Dokomajilar C, Sere Y, Rosenthal PJ, et al. Randomized Comparison of Amodiaquine plus Sulfadoxine-Pyrimethamine, Artemether-Lumefantrine, and Dihydroartemisinin-Piperaquine for the Treatment of Uncomplicated Plasmodium falciparum Malaria in Burkina Faso. Clin Infect Dis. 2007;45:1453–61.

10. Kamya MR, Yeka A, Bukirwa H, Lugemwa M, Rwakimari JB, Staedke SG, et al. Artemether-lumefantrine versus dihydroartemisinin-piperaquine for treatment of malaria: A randomized trial. PLoS Clin Trials. 2007;2.

11. Karunajeewa HA, Mueller I, Senn M, Lin E, Law I, Gomorrai PS, et al. A Trial of Combination Antimalarial Therapies in Children from Papua New Guinea. N Engl J Med. 2008;359:2545–57.

12. Yavo W, Faye B, Kuete T, Djohan V, Oga SA, Kassi RR, et al. Multicentric assessment of the efficacy and tolerability of dihydroartemisinin-piperaquine compared to artemether-lumefantrine in the treatment of uncomplicated Plasmodium falciparum malaria in sub-Saharan Africa. Malar J. 2011;10:1–8.

13. Khadime Sylla, Annie Abiola, Roger Clément Kouly Tine, Babacar Faye, Doudou Sow, Jean Louis Ndiaye, Magatte Ndiaye, Aminata Colé LO, Kuaku Folly LAN and OG. Monitoring the efficacy and safety of three artemisinin combinations therapies (ACT) in Senegal: Results from two years surveillance. Am J Trop Med Hyg. 2013;89 5 SUPPL. 1:251.

14. Smithuis F, Kyaw MK, Phe O, Win T, Aung PP, Oo APP, et al. Effectiveness of five artemisinin combination regimens with or without primaquine in uncomplicated falciparum malaria: An open-label randomised trial. Lancet Infect Dis. 2010;10:673–81.

15. Faye B, Ndiaye J-L, Ndiaye D, Dieng Y, Faye O, Gaye O. Efficacy and tolerability of four antimalarial combinations in the treatment of uncomplicated Plasmodium falciparum malaria in Senegal. Malar J. 2007;6:80.

16. Sirima SB, Ogutu B, Lusingu JPA, Mtoro A, Mrango Z, Ouedraogo A, et al. Comparison of artesunate-mefloquine and artemether-lumefantrine fixed-dose combinations for treatment of uncomplicated Plasmodium falciparum malaria in children younger than 5 years in sub-Saharan Africa: A randomised, multicentre, phase 4 trial. Lancet Infect Dis. 2016;16:1123–33.

17. Faye B, Ndiaye JL, Tine R, Sylla K, Gueye A, Lô AC, et al. A randomized trial of artesunate mefloquine versus artemether lumefantrine for the treatment of uncomplicated Plasmodium falciparum malaria in Senegalese children. Am J Trop Med Hyg. 2010;82:140–4.

18. Espié E, Lima A, Atua B, Dhorda M, Flévaud L, Sompwe EM, et al. Efficacy of fixed-dose combination artesunate-amodiaquine versus artemether-lumefantrine for uncomplicated childhood Plasmodium falciparum malaria in Democratic Republic of Congo: A randomized non-inferiority trial. Malar J. 2012;11:174.

19. Ndiaye JLA, Faye B, Gueye A, Tine R, Ndiaye D, Tchania C, et al. Repeated treatment of recurrent uncomplicated Plasmodium falciparum malaria in Senegal with fixed-dose artesunate plus amodiaquine versus fixed-dose artemether plus lumefantrine: A randomized, open-label trial. Malar J. 2011;10:237.

20. Offianan AT, Assi SB, Coulibaly A, N’guessan LT, Ako AA, Kadjo FK, San MK PL. Assessment of the efficacy of first-line antimalarial drugs after 5 years of deployment by the National Malaria Control Programme in Côte d’Ivoire. Open Access J Clin Trials. 2011;3:Pages 67—76.

21. Yeka A, Lameyre V, Afizi K, Fredrick M, Lukwago R, Kamya MR, et al. Efficacy and safety of fixed-dose artesunate-amodiaquine vs. artemether-lumefantrine for repeated treatment of uncomplicated malaria in Ugandan children. PLoS One. 2014;9:e113311.

22. Ndiaye J, Randrianarivelojosia M, Sagara I, Brasseur P, Ndiaye I, Faye B, et al. Randomized, multicentre assessment of the efficacy and safety of ASAQ – a fixed-dose artesunate-amodiaquine combination therapy in the treatment of uncomplicated Plasmodium falciparum malaria. Malar J. 2009;8:125.

23. Schramm B, Valeh P, Baudin E, Mazinda CS, Smith R, Pinoges L, et al. Tolerability and safety of artesunate-amodiaquine and artemether- lumefantrine fixed dose combinations for the treatment of uncomplicated Plasmodium falciparum malaria: Two open-label, randomized trials in Nimba County, Liberia. Malar J. 2013;12:250.

24. Faucher J-F, Aubouy A, Adeothy A, Cottrell G, Doritchamou J, Gourmel B, et al. Comparison of sulfadoxine-pyrimethamine, unsupervised artemether-lumefantrine, and unsupervised artesunate-amodiaquine fixed-dose formulation for uncomplicated plasmodium falciparum malaria in Benin: a randomized effectiveness noninferiority trial. J Infect Dis. 2009;200:57–65.
